# Genetic fate-mapping reveals surface accumulation but not deep organ invasion of pleural and peritoneal cavity macrophages following injury

**DOI:** 10.1038/s41467-021-23197-7

**Published:** 2021-05-17

**Authors:** Hengwei Jin, Kuo Liu, Juan Tang, Xiuzhen Huang, Haixiao Wang, Qianyu Zhang, Huan Zhu, Yan Li, Wenjuan Pu, Huan Zhao, Lingjuan He, Yi Li, Shaohua Zhang, Zhenqian Zhang, Yufei Zhao, Yanqing Qin, Stefan Pflanz, Karim E. I. Kasmi, Weiyi Zhang, Zhaoyuan Liu, Florent Ginhoux, Yong Ji, Ben He, Lixin Wang, Bin Zhou

**Affiliations:** 1 State Key Laboratory of Cell Biology, Shanghai Institute of Biochemistry and Cell Biology, Center for Excellence in Molecular Cell Science, Chinese Academy of Sciences, University of Chinese Academy of Sciences, Shanghai, China; 2 School of Life Science, Hangzhou Institute for Advanced Study, University of Chinese Academy of Sciences, Hangzhou, China; 3 School of Life Science and Technology, ShanghaiTech University, Shanghai, China; 4 Department of Cardiac Surgery, Zhongshan Hospital, Fudan University, Shanghai, China; 5 Shanghai Institute of Nutrition and Health, Chinese Academy of Sciences, Shanghai, China; 6 Boehringer Ingelheim Pharma GmbH & Co KG, Biberach an der Riss, Germany; 7 Shanghai Institute of Immunology, Department of Immunology and Microbiology, Shanghai Jiao Tong University School of Medicine, Shanghai, China; 8 Singapore Immunology Network, Agency for Science, Technology and Research, Singapore, Singapore; 9 The Collaborative Innovation Center for Cardiovascular Disease Translational Medicine, Nanjing Medical University, Nanjing, China; 10 Department of Cardiology, Shanghai Chest Hospital, Shanghai Jiaotong University, Shanghai, China

**Keywords:** Gene targeting, Imaging the immune system, Peritoneal macrophages, Acute inflammation

## Abstract

During injury, monocytes are recruited from the circulation to inflamed tissues and differentiate locally into mature macrophages, with prior reports showing that cavity macrophages of the peritoneum and pericardium invade deeply into the respective organs to promote repair. Here we report a dual recombinase-mediated genetic system designed to trace cavity macrophages in vivo by intersectional detection of two characteristic markers. Lineage tracing with this method shows accumulation of cavity macrophages during lung and liver injury on the surface of visceral organs without penetration into the parenchyma. Additional data suggest that these peritoneal or pleural cavity macrophages do not contribute to tissue repair and regeneration. Our in vivo genetic targeting approach thus provides a reliable method to identify and characterize cavity macrophages during their development and in tissue repair and regeneration, and distinguishes these cells from other lineages.

## Abstract (web summary)

Body cavity macrophages reside on the serous surfaces of organs and believed to participate in organ repair following injury. Here the authors show with a fate-mapping reporter system that these cells, although accumulate at the surfaces of injured liver or lung, don’t penetrate deeply into the tissue.

## Introduction

Macrophages are found in multiple tissues or organs with great functional diversity during development and during organ homeostasis, immunity, tissue repair, and regeneration^1^. Understanding this diversity and their contribution to pathophysiological processes may provide new therapeutic targets for many human diseases^2^. The long-held belief has been that tissue macrophages in the adult are continuously replenished by bone marrow-derived circulating monocytes^3,4^. In response to tissue injury and inflammation, monocytes from the circulation are recruited to the infected or damaged sites to neutralize and eliminate injurious stimuli^5^. These newly recruited monocytes differentiate into mature macrophages, where they promote tissue remodeling and repair^6^. Recently, a series of elegant lineage-tracing studies reveal that many adult tissue macrophages actually originate from resident embryonically-derived early progenitors cells, rather than from circulating monocytes, and that some of these resident macrophages can self-renew locally throughout adult life with minimal contribution from circulating monocytes^7–10^. For example, cardiac resident macrophages are key mediators of cardiac recovery by reducing inflammation and enhancing tissue repair^11^. Further, multiple cardiac macrophage subsets are found in the adult heart that is differentially derived and marked by various functions, including CCR2 expression, which distinguishes peripheral monocyte-derived macrophages from embryonically-derived resident macrophages^12–14^. Subsequent studies revealed that myocardial infarction induced replacement of resident macrophages with those from recruited monocytes, some of which adopted a cell fate nearly indistinguishable from resident macrophages^15^. These studies demonstrate that both circulating monocytes and tissue-resident macrophages are involved during tissue inflammation, repair, and regeneration.

In mammals, the peritoneal, pleural, and pericardial cavities, which are the three major cavities of adults, provide a mechanical buffer as well as lubrication for the movement of visceral organs. Recently, a new paradigm has emerged that proposes that distinct from monocyte-derived or tissue-resident macrophages, mature macrophages can be rapidly recruited from body cavities to the visceral organs, such as the liver and heart, to promote tissue repair after injury^16,17^. This so-called “wormhole migration” of cavity macrophages to visceral organs represents a new model for inflammatory cell recruitment^18^. Cavity macrophages express a high level of GATA6, which distinguishes them from other tissue-resident macrophages or circulating monocytes^19–21^. Cell transplantation assays using reporter mice reveal that GATA6^+^ cavity macrophages infiltrate visceral organs via the mesothelium, a single mesothelial cell layer that covers all visceral organs in the cavity^16,17^. Based on *Gata6* reporter activity, Kubes’ group reported that GATA6^+^ cavity macrophages invade deep into the injured liver and heart shortly after the initial insult via a nonvascular route, and many of them are detected greater than 500 µm from the organ surface^16,17^. Depletion of these cavity macrophages by clodronate treatment results in significant weight loss in a CCl_4_ model of liver fibrosis in mice^16^, while *Gata6* gene deletion using a *Lyz2-Cre* driver impaired the beneficial role of cavity macrophages in ameliorating cardiac fibrosis^17^. These *Gata6* reporter analyses and cell transplantation experiments provide the key evidence supporting a new model of inflammatory cell recruitment and contribution to tissue repair and regeneration. These exciting and highly influential findings may represent an entirely new field for macrophage biology and regenerative medicine, while also providing a potential therapeutic strategy to treat organ inflammation and to promote tissue repair. However, unlike the previous tissue-resident macrophage studies that utilized genetic lineage tracing to unravel the discrete origins of these cells, the new paradigm involving the recruitment of cavity macrophages during organ repair still lacks direct genetic lineage tracing evidence to support its conclusions.

Here, we report a novel dual recombinase-mediated genetic system that could be used for specifically labeling the cavity macrophages in vivo. Fate-mapping results show that cavity macrophages accumulate on the surface of the lung and liver without invading deep into the parenchyma after injuries. Furthermore, genetic ablation of cavity macrophages suggests their minimal functional contribution to tissue repair and regeneration. This study provides a specific genetic tool to better understand the precise roles of cavity macrophages in homeostasis, tissue repair, and regeneration.

## Results

### Generation of a cavity macrophage-specific mouse reporter for lineage tracing

Specific genetic targeting of endogenous cavity macrophages is critical to understand theirs in vivo cell fate and potential physiological functions in visceral organ inflammation and repair. However, a single genetic marker to distinguish these cells from other cell lineages has yet to be identified. The transcription factor GATA6 has been reported to be selectively expressed in resident cavity macrophages^19–22^ and distinguishes them from other tissue-resident macrophages and monocytes. But while GATA6 is specific for cavity macrophages among macrophage populations, it is also expressed in multiple non-macrophage cell lineages in various visceral organs, including hepatocytes, cardiomyocytes, lung epithelial cells, mesenchymal stromal cells, and other cell lineages of these organs^23–26^. This wide expression of GATA6 markedly undermines its utility to uniquely trace recruited cavity macrophages into visceral organs.

**Fig. 1:**
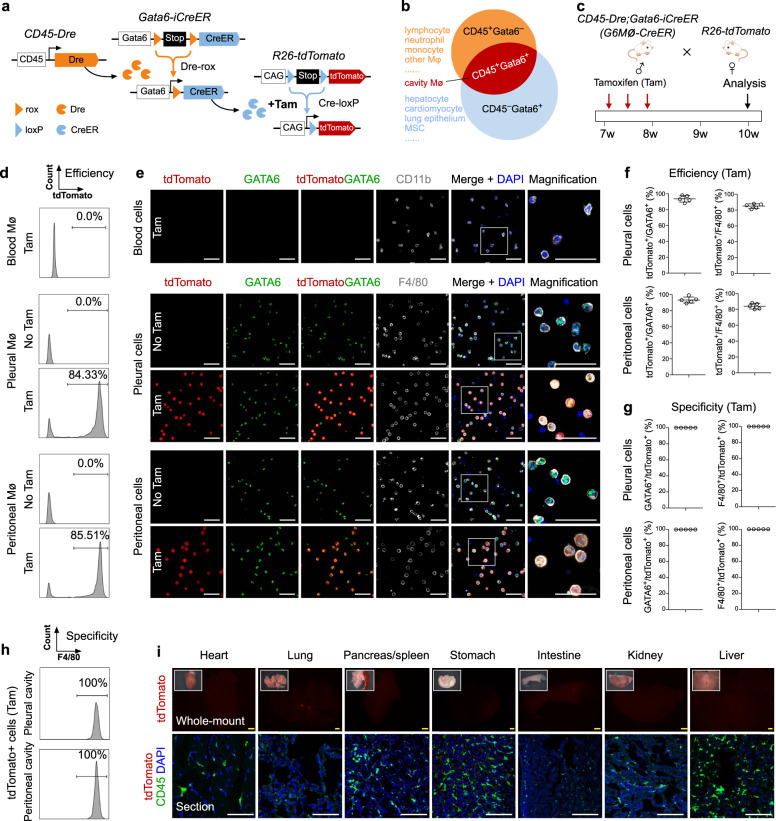
Generation and characterization of a genetic system for specific targeting of cavity macrophages. **a** Schematic figure showing *CD45-Dre* mediates Stop cassette removal from *Gata6-iCreER* and places CreER directly under Gata6 promoter. After tamoxifen (Tam), Cre-loxP recombination labels cells by tdTomato. **b** Intersectional genetics marks CD45^+^GATA6^+^ cells as tdTomato^+^. **c** Schematic figure showing experimental design using cavity macrophage tracing tool *CD45-Dre;Gata6-iCreER* (*G6Mø-CreER*). **d** Flow cytometric analysis of the percentage of tdTomato^+^ cells in macrophages from blood, pleural, and peritoneal cavity with or without Tam. **e** Immunostaining for tdTomato, CD11b, GATA6, F4/80 on dissociated cells from blood, pleural, or peritoneal cavity. Boxed regions are magnified. **f** Quantification of the percentage of tdTomato^+^ cells in GATA6^+^ or F4/80^+^ macrophages. Data are the mean ± SD; *n* = 5 mice per group. **g** Quantification of the percentage of GATA6^+^ or F4/80^+^ macrophages in tdTomato^+^ cells. Data are the mean ± SD; *n* = 5 mice per group. **h** FACS showing the percentage of tdTomato^+^ cells expressing F4/80. **i** Whole-mount epifluorescence images and immunostaining for CD45 and tdTomato shows no resident tdTomato^+^ macrophages in visceral organs. Each image is representative of 5 individual biological samples. Scale bars, yellow, 1 mm; white, 100 µm. Source data are provided as a Source Data file.

To allow for specific tracing of endogenous cavity macrophages, we developed a dual recombinase-mediated intersectional genetic lineage tracing system based on Cre-loxP and Dre-rox (Fig. 1a), which are orthogonal in their recombination^27–29^. In our design, the promoter of the gene encoding the hematopoietic cell marker CD45 was used to drive Dre recombinase, while the *Gata6* gene promoter was used to drive an inducible CreER (Fig. 1a). In this way, CD45 marker could be used to distinguish macrophages (as they are CD45^+^) from CD45^−^ organ cells (hepatocytes, cardiomyocytes, etc.); Gata6 marker could be used to distinguish cavity macrophages (as they are Gata6^+^) from recruited monocytes or resident-tissue macrophages (which are Gata6^−^). Thus, in theory, double-positive cells (i.e., CD45^+^Gata6^+^) would be cavity macrophages and thus be distinguished from tissue-resident macrophages or monocytes, as well as any other cell lineage, such as hepatocytes, cardiomyocytes, or lung epithelial cells (Fig. 1b). To make this system applicable for potential gene manipulation (i.e., knockout or over-expression) to facilitate functional studies, in addition to lineage tracing, we adopted a sequential intersectional genetic approach, by which Dre-mediated activation of CreER allows for subsequent targeting of any available loxP allele^30^. We anticipated that in CD45^+^ cells, the *CD45-Dre* driver would remove the rox-flanked transcriptional stop cassette, yielding a *Gata6-CreER* genotype after Dre-rox recombination. Subsequent tamoxifen (Tam) treatment would then enable a controlled CreER-mediated recombination event at the *R26-tdTomato* reporter, leading to constitutive expression of tdTomato in CD45^+^Gata6^+^ cells, and all their descendants, resulting in an indelible genetic marker that would allow for permanent and specific lineage tracing of cavity macrophages (Fig. 1a, b).

We first generated a *CD45-Dre* knock-in mouse line and crossed it with a strain bearing an *R26-rox-tdTomato* reporter^31^. We found that *CD45-Dre* specifically and efficiently targeted hematopoietic cells in the blood and spleen (Supplementary Fig. 1). We next generated *Gata6-rox-stop-rox-CreER* knock-in mice, a Dre-induced *Gata6-CreER* (*Gata6-iCreER*) line, by inserting a rox-stop-rox-CreER cassette into the *Gata6* gene locus to replace the endogenous translational start codon (Supplementary Fig. 2a). As there is a transcriptional stop cassette before CreER, Tam treatment alone would not be sufficient to induce tdTomato genetic labeling of GATA6^+^ cavity macrophages (Supplementary Fig. 2b, f). To test whether *Gata6-iCreER* could indeed label GATA6^+^ cells, we generated a *Gata6-CreER* mouse line by removing the transcriptional stop cassette from *Gata6-iCreER* by using *ACTB-Cre*^32^. Then by crossing *Gata6-CreER* with *R26-tdTomato* reporter mouse^33^, *Gata6-CreER* efficiently labeled cavity macrophages as tdTomato^+^ upon Tam treatment (Supplementary Fig. 3a, b). The generation of the inducible reporter line was critical as we found that the use of *Gata6-CreER* resulted in labeling of diverse cell types in multiple visceral organs, such as hepatocytes, epithelial cells, and cardiomyocytes (Supplementary Fig. 3c, d), which is consistent with previous reports on its broad expression pattern^23–26^. This broad genetic targeting of virtually all visceral organs and tissues excluded the direct use of the *Gata6-CreER* mouse for the study of cavity macrophages in visceral organ repair. We, therefore, took advantage of *CD45-Dre* to restrict the targeting domain of *Gata6-iCreER* in cavity macrophages.

For proof of principle, we crossed *CD45-Dre;Gata6-iCreER* (*G6Mø-CreER*) with *R26-tdTomato* and analyzed monocyte/macrophage labeling at two weeks after Tam treatment (Fig. 1c). FACS analysis and immunofluorescent (IF) staining revealed that the majority of pleural and peritoneal macrophages expressing CD11b and F4/80 were tdTomato^+^, while CD11b^+^ monocytes from blood mononuclear cells were tdTomato^−^ in Tam-treated *G6Mø-CreER;R26-tdTomato* mice (Fig. 1d–f). Virtually all tdTomato^+^ pleural and peritoneal cells (>99.5%) were GATA6^+^F4/80^+^ (Fig. 1g,h), indicating high specificity of *G6Mø-CreER* for labeling cavity macrophages. Of note, we did not detect any tdTomato^+^ cavity macrophages from *G6Mø-CreER;R26-tdTomato* mice without Tam treatment (No Tam, Fig. 1d, e), indicating no leakiness of *G6Mø-CreER* for cavity macrophage labeling. In all examined visceral organs collected from Tam-treated *G6Mø-CreER;R26-tdTomato* mice, we did not detect tdTomato expression in CD45^+^ cells, excluding ectopic labeling of resident macrophages or other hematopoietic cell lineages by *G6Mø-CreER* in these visceral organs (Fig. 1i). Taken together, the development of the *G6Mø-CreER* mouse allows for specific and efficient targeting of endogenous cavity macrophages.

### Resident cavity macrophages do not invade deep into the liver for its repair

**Fig. 2:**
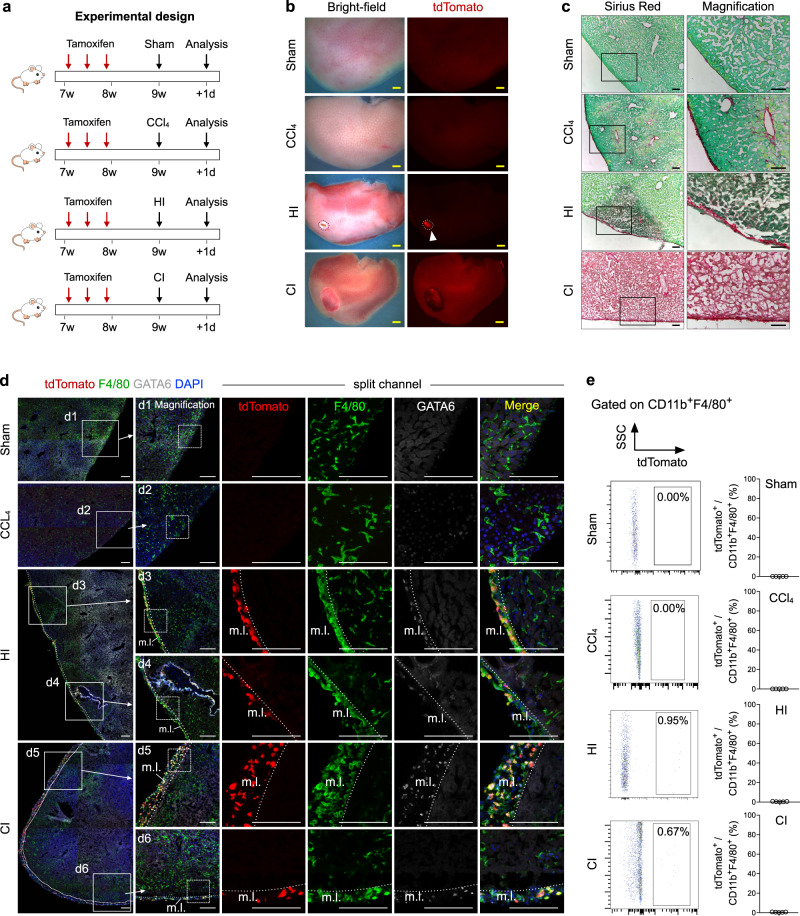
Peritoneal macrophages do not invade deep into the liver parenchyma after CCl_4_, HI, or CI treatment. **a** Schematic figure showing experimental strategy. HI heat injury, CI cryoinjury. **b** Whole-mount bright-field and fluorescent images of livers with different injury models. Arrowheads, injury site. Scale bars, 1 mm. **c** Sirius red staining of liver tissue sections from livers treated with CCl_4_, CI, HI, or sham operation. Boxed regions are magnified. Scale bars, 100 µm. **d** Immunostaining for tdTomato, GATA6, and F4/80 on the injured region of the liver section. m.l., mesothelial layer. Boxed regions are magnified. Scale bars, 100 µm. **e** FACS analysis and quantification of the percentage of F4/80^+^ macrophages expression tdTomato. Data are the mean ± SD; *n* = 5 mice per group. Each image is representative of five individual biological samples (**b**–**d**). Source data are provided as a Source Data file.

Previous studies have shown that GATA6^+^ cavity macrophages invade deep into the liver after CCl_4_- or heat-induced injury (HI, induced by touching a heated thermos probe to the liver surface). Further, transplantation of peritoneal macrophages from *LyzM-eGFP* donor mice into CCl_4_-injured host mice resulted in GFP^+^ cell migration across the mesothelium and penetration deep into the intrahepatic area^16^. To recapitulate the recruitment and invasion of GATA6^+^ cavity macrophages without a cell transplantation approach, we performed the same injury models of CCl_4_ treatment and HI, and also included additional cryoinjury (CI) and acetaminophen (APAP) injury models in *G6Mø-CreER;R26-tdTomato* mice (Fig. 2a and Supplementary Fig. 5a). For sham control, we opened the abdomen and closed it without any further manipulation. One week after Tam injection, mice were challenged with different injury models and livers were collected shortly after treatment as previously indicated^16^. While we could hardly detect any noticeable tdTomato^+^ signals from whole-mount fluorescent livers collected from sham, CCl_4_- and APAP-treated mice, we did observe bright tdTomato^+^ regions in the livers from the HI and CI models (Fig. 2b and Supplementary Fig. 5b). Sirius Red staining of liver sections showed a greater fibrotic response in the CCl_4_- and APAP-treated groups compared to the Sham control (Fig. 2c and Supplementary Fig. 5c), consistent with previous studies reporting that a single dose of CCl_4_ or APAP results in severe necro-inflammatory injury peaking at day 1 after injury^16,34^. We noticed that in the HI model the injured liver region exhibited thickened mesothelial cell layers that covered the surface of livers, while in the CI model injured liver regions showed pronounced Sirius Red-positive staining, suggestive of severe necrosis (Fig. 2c).

We next examined tdTomato expression in livers of the above injury models. Immunostaining of liver sections for tdTomato, F4/80, and GATA6 revealed no accumulation of tdTomato^+^ cavity macrophages inside liver parenchymal tissue after CCl_4_ or APAP treatment (Fig. 2d and Supplementary Fig. 5d), in contrast to previously reported data showing that a substantial number of transplanted LysM-GFP^+^ peritoneal cells infiltrated over 500 µm into the intrahepatic area^16^. FACS analysis confirmed almost no tdTomato^+^ macrophages in Sham, CCl_4_-or APAP-treated livers (Fig. 2e and Supplementary Fig. 5e). In injured regions of the liver from both the HI and CI models, we did notice an accumulation of tdTomato^+^ cavity macrophages, but virtually all of the cells were on the surface of thickened mesothelial layers (m.l.) without any noticeable deep invasion into the liver parenchyma (Fig. 2d). FACS analysis of cells collected from the tdTomato-bright regions (i.e., the injury sites) of the livers from the HI or CI models revealed less than 1% of macrophages were tdTomato^+^ (Fig. 2e). During late phase after injury, immunostaining and FACS analysis of CCl_4_-treated livers further confirmed tdTomato^+^ macrophages minimally invaded deep into the injured tissues at any detected time points (Supplementary Fig. 6). To further investigate whether transplanted cavity macrophages could invade deep into the liver after injuries, peritoneal macrophages from the *G6Mø-CreER;R26-tdTomato* mice were collected and transferred to the wild-type mice before HI- and CCl_4_-induced injuries. Immunostaining and FACS analysis of injured areas showed no evidence for the migration of tdTomato^+^ macrophages into the parenchymal tissues (Supplementary Fig. 7). In addition to the acute injury, we also performed CCl_4_-induced chronic liver injury on *G6Mø-CreER;R26-tdTomato* mice, and found cavity macrophages minimally invaded visceral organs for tissue repair and regeneration even in chronic injury models (Supplementary Fig. 8). Taken together, genetic fate mapping of endogenous cavity macrophages revealed that they did not invade deep into the liver after CCl_4_-, HI-, or CI-induced injuries.

### Pleural cavity macrophages minimally contribute to lung repair

**Fig. 3:**
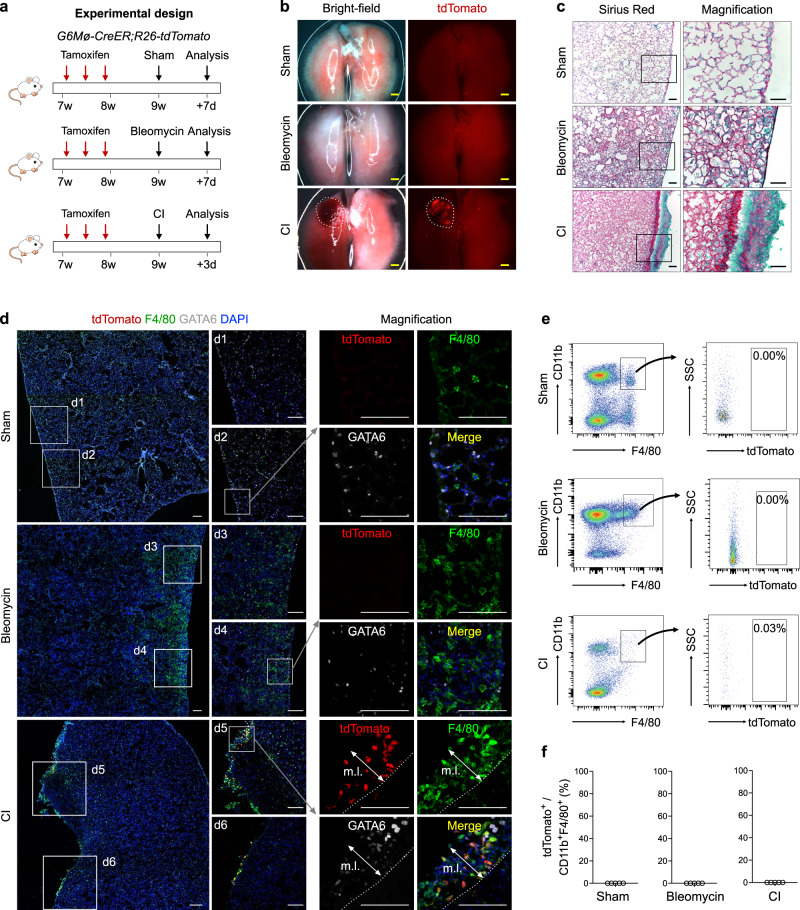
Pleural macrophages do not invade deep into the lung parenchyma after bleomycin treatment or CI. **a** Schematic figure showing experimental strategies. CI cryoinjury. **b** Whole-mount bright-field and fluorescent images of lungs after different injuries. Dotted line, CI region. Scale bars, 1 mm. **c** Sirius red staining of lung tissue sections after bleomycin or cryoinjury in lungs. Boxed regions are magnified. Scale bars, 100 µm. **d** Immunostaining for tdTomato, GATA6, and F4/80 on injured regions of lungs. m.l., mesothelial layer. Boxed regions are magnified. Scale bars, 100 µm. **e**, **f** FACS and quantification analysis of the percentage of macrophages expressing tdTomato from injury regions of the lung. Data are the mean ± SD; *n* = 5 mice per group. Each image is representative of five individual biological samples (**b**–**d**). Source data are provided as a Source Data file.

To further explore whether repair of other visceral organs may involve cavity macrophages, we next studied if pleural cavity macrophages could invade into the lung for its repair and regeneration after injury. We used a lung alveolar-injury model induced by bleomycin, CI or lipopolysaccharide (LPS) to examine the contribution of cavity macrophage in lung repair (Fig. 3a and Supplementary Fig. 5f). Whole-mount fluorescence images of lungs collected from *G6Mø-CreER;R26-tdTomato* mice showed a tdTomato^+^ region in the lung from the CI mice, and rare sporadic tdTomato^+^ cells on the surface of the lungs from Sham, bleomycin- or LPS-treated mice (Fig. 3b and Supplementary Fig. 5g). Sirius Red staining on lung sections showed a significant increase of tissue fibrosis in the bleomycin, CI and LPS groups, compared with the Sham control (Fig. 3c and Supplementary Fig. 5h). Of note, the surface of the lung in the CI group exhibited thickened mesothelial cell layers enriched for fibrosis (Fig. 3c). Next, we performed immunostaining of lung sections for tdTomato, F4/80 and GATA6, and we found markedly greater staining for F4/80^+^ macrophages in the bleomycin- and LPS-treated mice compared with that of the Sham control (Fig. 3d and Supplementary Fig. 5i). Notably, the GATA6^+^ cells detected in the lung parenchyma were not macrophages, as they did not express F4/80 (Fig. 3d). In the lungs of CI-treated mice, tdTomato^+^ cavity macrophages were mainly restricted to the thickened mesothelial layers on the surface rather than invasion deep into the lungs (Fig. 3d). We next performed FACS analysis of CD11b^+^F4/80^+^ cells collected from injured regions of the lungs from the CI, bleomycin- and LPS-treated mice, and found that a negligible percentage of macrophages (~0.03%) expressed tdTomato (Fig. 3e,f and Supplementary Fig. 5j). These data demonstrate that pleural cavity macrophages minimally invade the lung, suggesting that they do not contribute to lung repair and regeneration.

### Ablation of cavity macrophages do not significantly affect visceral organ repair

**Fig. 4:**
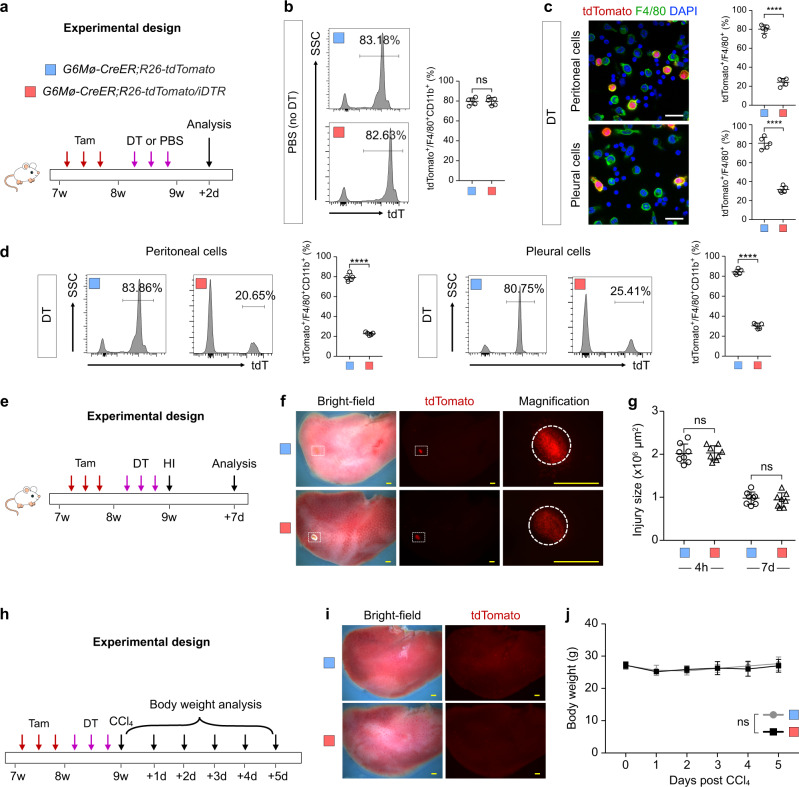
Genetic ablation of cavity macrophages did not affect liver repair after injury. **a** Schematic figure showing the experimental design. DT diphtheria toxin. **b** Flow cytometric and quantification analysis of the percentage of CD11b^+^F4/80^+^ macrophages expressing tdTomato from peritoneal cavity after PBS treatment (no DT). Data are the mean ± SD; *n* = 5 mice per group; ns nonsignificant. **c** Immunostaining for tdTomato and F4/80 on dissociated cells from the peritoneal or pleural cavity after DT treatment of the *G6Mø-CreER;R26-tdTomato/iDTR* mice. Quantification of the percentage of F4/80^+^ macrophages expressing tdTomato. Data are the mean ± SD; *n* = 5 mice per group; ****P < 0.0001. Each image is representative of five individual biological samples. **d** Flow cytometric and quantification analysis of the percentage of CD11b^+^F4/80^+^ macrophages expressing tdTomato in the pleural cavity, and peritoneal cavity, respectively. Data are the mean ± SD; *n* = 5 mice per group; ****P < 0.0001. **e** Schematic figure showing the experimental design. HI heat injury. **f** Whole-mount bright-field and fluorescence images of livers at 7 days after HI. Boxed regions are magnified; circles, heat injury site. Each image is representative of eight individual biological samples. **g** Quantification of injury size from 4 h to 7 days after heart injury. Data are the mean ± SD; *n* = 8 mice per group; ns nonsignificant. **h** Schematic figure showing the experimental design. **i** Whole-mount bright-field and fluorescence images of livers at 5 days after CCl_4_ treatment. Each image is representative of five individual biological samples. **j**, Quantification of body weight at different days after CCl_4_ treatment. Data are the mean ± SD; n = 5 mice per group; ns, non-significant. Scale bars: yellow, 1 mm; white, 100 µm. *P* value was calculated by unpaired two-sided Student’s *t* test (**b**–**d**, **g**) or two-way ANOVA coupled with multiple comparisons (**j**). Source data are provided as a Source Data file.

It has been previously reported that in a CCl_4_ model of organ fibrosis that clodronate liposome-mediated depletion of cavity macrophages results in a greater loss of body weight compared with control mice^16^. Whether clodronate had any side effect on liver hepatocytes or other macrophage populations during recovery from injury remained unclear. To specifically dissect the reparative function of GATA6^+^ cavity macrophages after liver injury, we crossed *G6Mø-CreER;R26-tdTomato* with the inducible diphtheria toxin receptor (DTR) mouse line *R26-iDTR*^35^ to allow selective ablation of GATA6^+^ cavity macrophages by subsequent DT treatment^36,37^ (Fig. 4a). Phosphate-buffered saline (PBS) injection in the *G6Mø-CreER;R26-tdTomato/iDTR* mice did not result in any significant loss of labeled cavity macrophages (Fig. 4b). When we are immunostained for tdTomato and F4/80 to identify cavity macrophages, we found significantly lower numbers of tdTomato^+^ macrophages from the *G6Mø-CreER;R26-tdTomato/iDTR* mice compared to the *G6Mø-CreER;R26-tdTomato* mice after DT treatment (Fig. 4c). Further, when we performed FACS analysis, we confirmed a significantly lower percentage of tdTomato^+^ macrophages among peritoneal or pleural macrophages (Fig. 4d), indicating successful genetic ablation of the majority of tdTomato^+^ macrophages. After Tam and DT treatments, we then induced HI and collected liver samples at 4 h and 7 days after injury (Fig. 4e). There were no significant differences in the injury size between *G6Mø-CreER;R26-tdTomato/iDTR* mice and *G6Mø-CreER;R26-tdTomato* mice after DT treatment (Fig. 4f, g). Furthermore, in the CCl_4_ model, in which we examined body weight every day for 5 days after treatment (Fig. 4h), we did not detect any morphological changes in the liver (Fig. 4i) nor differences in body weight (Fig. 4j) between *G6Mø-CreER;R26-tdTomato/iDTR* mice and *G6Mø-CreER;R26-tdTomato* mice. To further confirm the impact of cavity macrophages in CCl_4_-induced liver injury, we measured the levels of alanine aminotransferase (ALT) and aspartate aminotransferase (AST) in serum at a different time point after CCl_4_ treatment (Supplementary Fig. 9a–c). Genetic ablation of cavity macrophages did not significantly impact the ALT or AST level, or severity of tissue fibrosis, compared with controls treated with CCl_4_ (Supplementary Fig. 9d–g). The above results suggested that ablation of cavity macrophages did not significantly affect visceral organ repair after injury.

### Knockout of *Gata6* in cavity macrophages has no impact on visceral organ repair

**Fig. 5:**
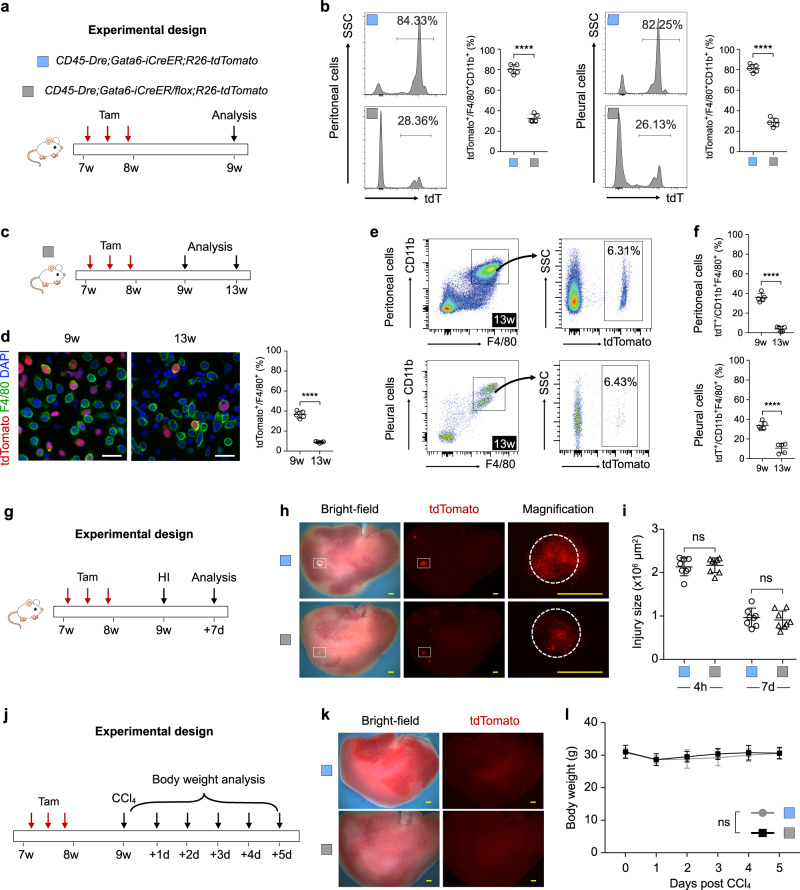
Gata6 deletion in cavity macrophages did not affect injury repair in the liver. **a** Schematic figure showing the experimental design. **b** Flow cytometric and quantification analysis of the percentage CD11b^+^F4/80^+^ macrophages expressing tdTomato from the pleural and peritoneal cavity. Data are the mean ± SD; *n* = 5 mice per group; ****P < 0.0001. **c** Schemati**c** figure showing the experimental design. **d** Immunostaining for tdTomato and F4/80 on dissociated cells from peritoneal cavity at 9 weeks and 13 weeks after TAM. Data are the mean ± SD; *n* = 5 mice per group. ****P < 0.0001. Each image is representative of five individual biological samples. **e**, **f** Flow cytometric analysis and quantification of the percentage of CD11b^+^F4/80^+^ macrophages expressing tdTomato. Data are the mean ± SD; *n* = 5 mice per group; ****P < 0.0001. **g** Schematic figure showing the experimental design. HI heat injury. **h** Whole-mount bright-field and fluorescence images of livers collected at 7 days after heart injury. Boxed regions are magnified; circles, heat injury site. Each image is representative of eight individual biological samples. **i** Quantification of injury size at 4 h and 7 days post HI. Data are the mean ± SD; *n* = 8 mice per group; ns nonsignificant. **j** Schematic figure showing the experimental design. **k** Whole-mount bright-field and fluorescence images of livers collected at 5 days after injury. Each image is representative of five individual biological samples. **l** Quantification of body weight at different days after CCl_4_ treatment. Data are the mean ± SD; *n* = 5 mice per group; ns, non-significant. Scale bars: yellow, 1 mm; white, 100 µm. *P* value was calculated by unpaired two-sided Student’s *t* test (**b**, **d**, **f**, **i**) or two-way ANOVA coupled with multiple comparisons (**l**). Source data are provided as a Source Data file.

GATA6 has been reported to play an essential role in cavity macrophage-specific function, phenotype, and proliferation^20^. To address whether impaired cavity macrophage function would markedly influence tissue repair, we crossed *CD45-Dre;Gata6-iCreER;R26-tdTomato* mice with those harboring a *Gata6-flox* allele and generated *CD45-Dre;Gata6-iCreER/flox;R26-tdTomato* mice. We then treated the newly generated mice with Tam three times between weeks 7 and 8 of age to delete *Gata6* and to genetically label cavity macrophages, followed by FACS analysis 1 week later (Fig. 5a). Upon FACS analysis, we observed a significantly lower percentage of tdTomato^+^ macrophages in both the peritoneal and pleural cavities of *CD45-Dre;Gata6-iCreER/flox;R26-tdTomato* mice compared with those in *CD45-Dre;Gata6-iCreER;R26-tdTomato* control mice (Fig. 5b). We next followed the TAM-treated mice for another four weeks after the initial analysis (Fig. 5c). We observed a further reduction of tdTomato^+^ cavity macrophages over time (Fig. 5d–f), which is consistent with previous studies showing that *Gata6* deficiency results in dysregulated peritoneal macrophage proliferative renewal^20,22^. In a separate cohort of TAM-treated mice we injured the liver via HI and collected tissues for analysis 1-week later (Fig. 5g). By whole-mount fluorescence imaging of the livers we found no significant difference in injury size between *CD45-Dre;Gata6-iCreER/flox;R26-tdTomato* mice and *CD45-Dre;Gata6-iCreER;R26-tdTomato* control mice (Fig. 5h, i). In yet another cohort of TAM-treated mice we also induced injury via CCl_4_ treatment and examined their body weight every day afterward for 5 days (Fig. 5j). We did not observe any difference in the gross morphology of the liver between the two groups of mice (Fig. 5k). Nor did we find any significant difference in body weight (Fig. 5l). Next, the serum ALT and AST levels, and fibrosis were evaluated at the indicated times after CCl_4_ stimulation, and we found that no significant difference between *CD45-Dre;Gata6-iCreER/flox;R26-tdTomato* mice and *CD45-Dre;Gata6-iCreER;R26-tdTomato* control mice (Supplementary Fig. 9). The combined results above indicate that impaired function of cavity macrophages did not have a noticeable effect on the organ repair.

### *Gata6* heterozygosity does not affect cavity macrophage function

Considering that *Gata6* is functionally essential for the maintenance of cavity macrophage number and function^20,22^, the lack of invasion of *Gata6*^+^ cavity macrophages in our system could be due to the *Gata6* heterozygosity, in case one allele of which was unintentionally disrupted by *Gata6-iCreER* knockin. To examine if this possibility was indeed a confounding factor in explaining our results, we examined the number, phagocytosis, and polarization genes of *Gata6*^*+/−*^ cavity macrophages in comparison with wild-type controls (Supplementary Fig. 4a). By immunostaining and FACS analysis, we did not find any difference in the number of GATA6^+^ macrophages between *Gata6*^*+/−*^ and wild-type mice (Supplementary Fig. 4b, c). Further, immunostaining and FACS analysis of fluorescent beads phagocytosed by macrophages showed no significant difference between cavity macrophages collected from *Gata6*^*+/−*^ and wild-type mice (Supplementary Fig. 4d, e). Quantitative real-time polymerase chain reaction (qRT-PCR) analysis of polarization genes for M1-like and M2-like macrophages revealed no significant difference in expression between the *Gata6*^*+/−*^ and wild-type *Gata6*^*+/+*^ groups (Supplementary Fig. 4f). The above data indicate that cavity macrophages from heterozygous Gata6 mice have normal function.

### Generation of *Gata6-iCreER2* for tracing cavity macrophages without *Gata6* loss

**Fig. 6:**
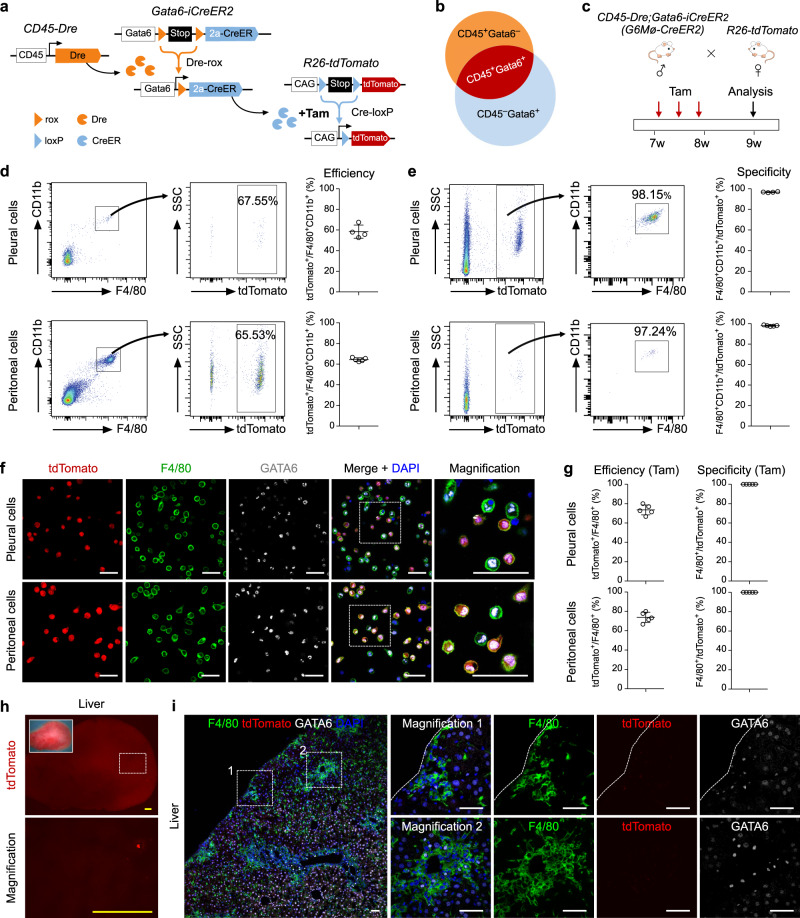
Generation and characterization of *Gata6-iCreER2* for fate mapping of cavity macrophages. **a** Schematic figure showing experimental strategy. *Gata6-iCreER2* uses a knock-in strategy that maintains endogenous Gata6 gene expression. **b** Intersectional genetics marks CD45^+^GATA6^+^ cells as tdTomato. **c** Schematic figure showing experimental design using a second cavity macrophage CreER line (*G6Mø-CreER2*). **d**, **e** Flow cytometric analysis of the percentage of tdTomato^+^ cells in macrophages from the pleural and peritoneal cavity (left panel); and the percentage of F4/80^+^ macrophages in tdTomato^+^ cells from the pleural and peritoneal cavity (right panel). For pleural cells, data are the mean ± SD; *n* = 4 mice per group. For peritoneal cells, data are the mean ± SD; *n* = 5 mice per group. **f** Immunostaining for tdTomato, GATA6, and F4/80 on dissociated cells from the pleural or peritoneal cavity. Boxed regions are magnified. **g** Quantification of the percentage of F4/80^+^ macrophages expressing tdTomato, or the percentage of tdTomato^+^ cells expressing F4/80. Data are the mean ± SD; *n* = 5 mice per group. **h** Whole-mount epifluorescence images of the liver after CCl_4_ injury. The boxed region is magnified. **i** Immunostaining for tdTomato, GATA6, and F4/80 on the injured region of livers. Dotted lines indicate the surface of tissue sections. Boxed regions are magnified. Each image is representative of five individual biological samples (**f**, **h**, **i**). Scale bars, yellow, 1 mm; white, 100 µm. Source data are provided as a Source Data file.

Finally, we generated a new allele of *Gata6-iCreER2* in which we inserted rox-Stop-rox-P2A-CreER into the 3′ UTR of the *Gata6* gene, thus maintaining the normal structure of the coding sequence of the endogenous gene (Fig. 6a). We then crossed mice harboring this new allele of *Gata6-iCreER2* with those carrying *CD45-Dre* for targeting the CD45^+^Gata6^+^ cell population (Fig. 6b). We then crossed *CD45-Dre;Gata6-iCreER2* (*G6Mø-CreER2*) mice with *R26-tdTomato* mice to enable genetic lineage tracing of cavity macrophages after Tam treatment (Fig. 6c). FACS analysis revealed both high efficiency and specificity of the *G6Mø-CreER2* allele in the labeling of pleural and peritoneal cavity macrophages (Fig. 6d, e). Immunostaining of isolated cavity cells for tdTomato, F4/80 and GATA6 confirmed that the majority of cavity macrophages were genetically labeled, and almost all labeled cells were macrophages (Fig. 6f, g).

**Fig. 7:**
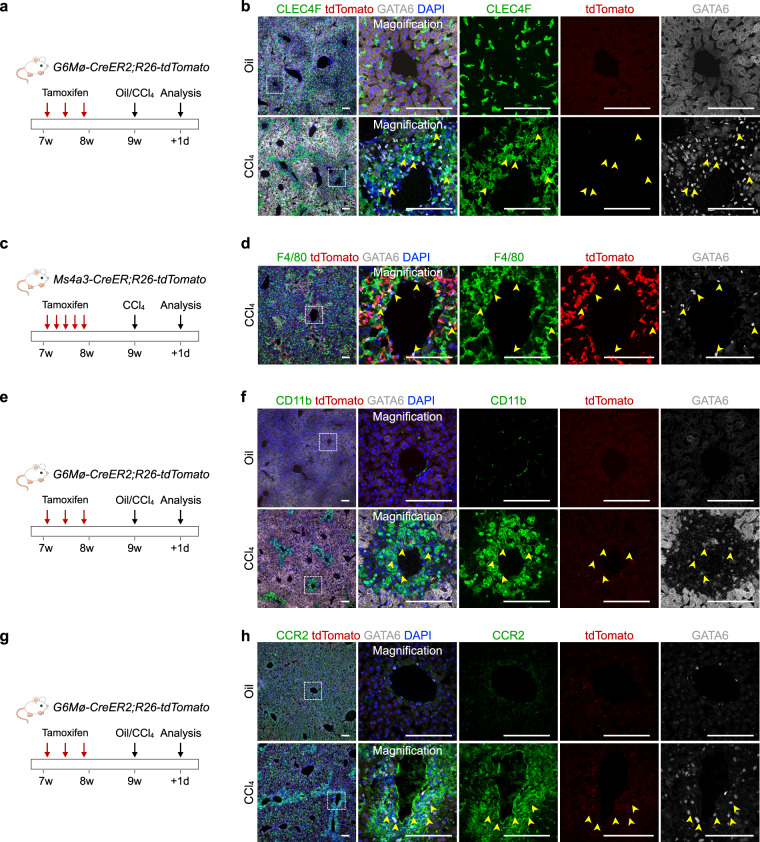
GATA6^+^tdTomato^−^ cells in the CCl_4_-treated livers are mainly resident Kupffer cells. **a** Schematic figure showing experimental strategy. **b** Immunostaining for tdTomato, GATA6, and CLEC4F on liver sections. **c** Schematic figure showing experimental strategy. **d** Immunostaining for tdTomato, GATA6, and F4/80 on the injured region of the liver section. **e** Schematic figure showing experimental strategy. **f** Immunostaining for tdTomato, GATA6, and CD11b on liver sections. **g** Schematic figure showing experimental strategy. **h** Immunostaining for tdTomato, GATA6, and CCR2 on liver sections. Scale bars, 100 µm. For **b**, **d**, **f**, **h** arrowheads indicate GATA6^+^ cells after injury. Boxed regions are magnified. Each image is representative of five individual samples.

We then used Tam-treated *G6Mø-CreER2;R26-tdTomato* mice for CCl_4_-induced injury and examined the recruitment of tdTomato^+^ cavity macrophages into the liver. By whole-mount fluorescence imaging of livers from CCl_4_-treated mice, we found very few tdTomato^+^ cells on the surface of the liver (Fig. 6h). Likewise, by immunostaining liver sections for F4/80, tdTomato, and GATA6 we found no tdTomato^+^ macrophages in the parenchyma of injured livers (Fig. 6i). Of note, GATA6 was also detected in the injured liver, indicating its broad expression in non-cavity macrophages of the liver after CCl_4_ injury (Fig. 6i). Next, immunostaining for Kupffer cells marker CLEC4F on liver sections of CCl_4_-treated mice indicated that GATA6^+^tdTomato^–^ cells were mainly resident Kupffer cells (Fig. 7a, b). Furthermore, by using *Ms4a3-CreER;R26-tdTomato* mice, we could trace the monocyte-derived cells under CCl_4_ administration^38^. Immunostaining for F4/80, tdTomato, and GATA6 on liver sections suggested that most GATA6^+^tdTomato^–^ did not come from monocytes (Fig. 7c, d). Immunostaining for CD11b and CCR2 on liver sections of *G6Mø-CreER2;R26-tdTomato* mice showed significantly increased expression levels in GATA6^+^ macrophages in injured areas (Fig. 7e–h). These data independently support the notion that cavity macrophages contribute minimally, if at all, to tissue repair and regeneration after pharmacologically induced liver injury.

## Discussion

**Fig. 8:**
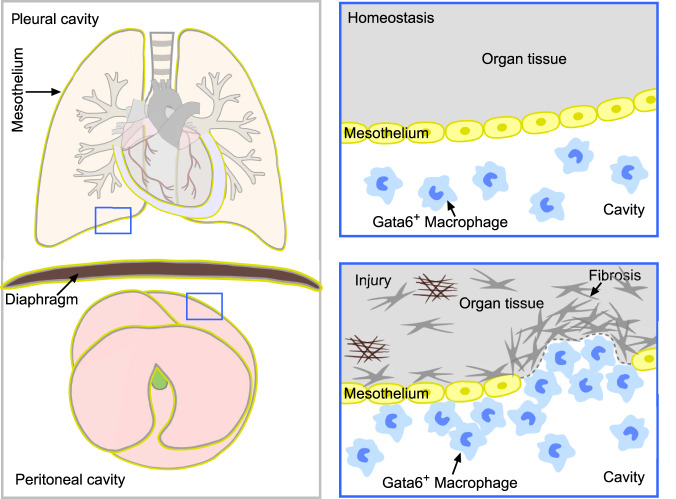
Pleural and peritoneal cavity macrophages minimally invade visceral organs after injuries. *Gata6*^*+*^ cavity macrophages are located in peritoneal and pleural cavities, which are separated by mesothelium that wraps visceral organs such as lung, and liver. After injuries, cavity macrophages are recruited to the surface of visceral organs, but they do not infiltrate into the parenchyma of organs. Nor do they play a functional role in tissue repair and regeneration.

In this study, we generated a genetic system to target cavity macrophages. By employing dual recombinase-mediated genetic lineage tracing, we specifically and efficiently traced GATA6^+^ cavity macrophages during tissue homeostasis and injuries. In contrast to previous reports showing deep invasion and a significant contribution of these cells to the functional recovery of visceral organs after injury^16,17^, our genetic fate mapping approaches reveal that cavity macrophages minimally invaded visceral organs after injury and contributed negligibly to the repair and regeneration of the liver or lung (Fig. 8). Genetic-based cell ablation and *Gata6* gene deletion in cavity macrophages did not significantly affect the repair of injured organs. Our study raises concerns about the conclusions of previously reported results based on Gata6 gene activity and cell transplantation for cavity macrophage study^16,17^. Whether cavity macrophages have additional roles in visceral organ repair other than direct invasion remains to be explored further in the future.

Currently, there are no infallible means of tracking cell fate in any organ system. However, genetic fate mapping approaches provide the strongest level of spatiotemporal resolution, hence the level of scientific evidence for in vivo cell origin and fate in multiple fields^39–44^. The strength of genetic lineage tracing technology is that it allows for the permanent and irreversible marking of cells and all their descendants in vivo, no matter where they migrate and how they might evolve during tissue homeostasis or injuries. Further, such a genetic approach allows for the tracing of cells under more physiological conditions that avoid the stressful conditions induced by cell transplantation and in vitro cell manipulation. We, therefore, chose to use a genetic lineage tracing approach to assess cell fate of cavity macrophages in vivo, which provides an orthogonal approach significantly different from the methods used in previous studies^16,17^, which relied mainly on measuring gene activity of cavity macrophage markers and cell transplantation for tracing these cells in visceral organs^16^. In particular, F4/80 staining was used for determining the location of cavity macrophages, which has the caveat of not knowing the origin(s) of these cells^16^. The expression map, e.g., GATA6 expression, is not reliable as gene activity can change depending on the surrounding conditions, due to diverse origins of macrophages, macrophage plasticity, and their dynamic microenvironment^1,45–47^. The cell transplantation experiments, such as the transfer of peritoneal cells from *LysM-eGFP* mice^16^ or *Gata6-Venus* bone marrow cells transplanted into irradiated mice^17^, are important to define the contribution of cavity macrophages in visceral organs. However, a general caveat to the cell transplantation approach is that the process may represent an artificial state or extraordinary condition that forces these transplanted cells to adopt potentially enhanced functions for which they usually do not display under physiological conditions. Numerous studies have documented the discrepancy of conclusions regarding the interpretation of cell fate based on a cell transplantation approach vs. and one based on genetic fate mapping. For example, hair follicle bulge stem cells give rise to all epidermal lineages upon cell transplantation but only to hair follicle regeneration under physiological conditions^48^. As another example, in contrast to the cell plantation study showing that life-long blood cell production is driven by a small number of multipotent hematopoietic stem cells^49^, a subsequent in situ genetic tracing study revealed that steady-state blood production is actually maintained by the successive recruitment of thousands of clones^50^. In addition, while single multipotent stem cells have the capacity to produce sufficient differentiated progeny to constitute an entire functional mammary gland after transplantation^51^, genetic lineage tracing suggests that unipotent rather than multipotent stem cells contribute to the maintenance of luminal and basal mammary epithelial cells in the adult^52^. Finally, a cell transplantation assay identifies c-Kit^+^ multipotent lung stem cells^53,54^, which was subsequently called into question by a genetic lineage tracing study^54^. These compelling examples provide a reasonable explanation as to why the conclusion of our current lineage tracing study may differ from those based on a cell transplantation assay to study the role of cavity macrophages in tissue repair; they also highlight the value of genetic lineage tracing experiments to further interrogate findings from the transplantation study setting.

Taken together, our work here, based on a genetic approach to specifically trace and manipulate cavity macrophages in vivo, suggests that cavity macrophages minimally invade visceral organs after injury to contribute to tissue repair and regeneration, and thus appear to contradict the conclusions of previous studies in this regard^16,17^. Even so, it could be possible that under other circumstances than those tested here cavity macrophages could be activated to invade deep into organs and play an essential role during the repair. But such a paradigm needs to be revisited by other independent groups and by different injury models in the future.

## Methods

### Mice

All animal protocols used in this study were approved by the experimental animal facility which has been accredited by the Institutional Animal Care and Use Committee (IACUC) of the State Key Laboratory of Cell Biology, Shanghai Institute of Biochemistry and Cell Biology, Center for Excellence in Molecular Cell Science, University of Chinese Academy of Sciences, Chinese Academy of Sciences. The previously described mouse strains used in this study included *R26-iDTR, CAG-Dre*, *R26-tdTomato*, *R26-rox-tdTomato*, *Gata6*^*flox*^ and *Ms4a3-CreER* mice^35,38,55–57^. The *R26-iDTR* and *Gata6*^*flox*^ mice were originally obtained from The Jackson Laboratory and the stock# were jax007900 and jax008196, respectively. The *CD45-Dre* knock-in mouse line was generated by homologous recombination using CRISPR/Cas9. The cDNA encoding Dre recombinase was inserted into CD45 gene locus to replace the endogenous translational start codon and was followed by a transcriptional stop polyA sequence. The *Gata6-rox-stop-rox-CreER* (*Gata6-iCreER*) knock-in mouse line was constructed by inserting a *rox-stop-rox-CreER-WPRE* (Woodchuck hepatitis virus regulatory element)*-polyA* cassette into the *Gata6* gene, replacing the endogenous translational start codon. The *Gata6-iCreER* strain was generated by conventional homologous recombination using ES cells with neomycin for selection. These mouse lines were generated by Shanghai Biomodel Organism Science and Technology Development, Shanghai, China. All mouse lines were maintained in a C57BL/6 genetic background. Mice were housed in accordance with the regulations on mouse welfare and ethics of the Institute of Biochemistry and Cell Biology in groups with 12-h dark-light cycles and had free access to food and water. Both male and female mice of the adult stage beginning at 7 weeks old were included in the study. Tamoxifen (Sigma, T5648) was dissolved in corn oil (20 mg/mL) and administered by gavage at the indicated time points. For DT administration, mice were injected intraperitoneally with 10 ng/g body weight DT (dissolved in PBS; Sigma, D0564-1MG). No obvious adverse side effects of DT were detected when administered to the control and *R26-iDTR* mice. All mice were kept in group housing (2–5 mice per cage) in a specific pathogen-free facility with controlled environmental conditions of temperature (20–25 °C), humidity (30–70%), and lighting (a 12-h light/dark cycle).

### Euthanasia for the animal experiments

For mice, carbon dioxide (CO_2)_ was used for euthanasia in an appropriate euthanasia box. After exposure to CO_2_, a careful assessment was needed to be done to confirm no signs of life in mice, like breathing and heartbeat. Then the mice were removed for the following steps.

### Genomic PCR

For genotyping of mice, the biopsies (tail tips) were collected approximately 7 days after birth. These biopsies were digested by tail lysis buffer (300 μl) with proteinase K (5 μl, Roche, 3115852001) at 55 °C for at least 4 h, and 600 μl absolute EtOH was added for centrifugation at 10,656*g* for 2 min. The supernatant was discarded and 600 μl 70% EtOH was added for centrifugation at 10,656*g* for 2 min. Afterward, the liquid was discarded and the DNA air-dried. Totally, 200 μl of distilled water was added to dissolve the DNA, for PCR amplification. Primers and protocol for PCR are available upon request.

### Tissue whole-mount fluorescence microscopy

Collected visceral organs were washed in PBS and placed on agar in the required direction for whole-mount bright-field and fluorescence images by using a Zeiss stereoscope (AxioZoom V16). To determine the magnification of specific regions, we used the automated z-stack images acquired with a Zeiss stereoscope (AxioZoom V16).

### Tissue collection and IF staining and imaging

After killing of the mice, tissues were fixed in 4% paraformaldehyde (PFA, Sigma, P6148) for no more than 1 h at 4 °C. After fixation, the tissues were rinsed in PBS and incubated in 30% sucrose at 4 °C overnight. Then, the tissues were embedded in OCT (Sakura, 4583) medium in the required direction and stored at −80 °C. Sections were cut at a thickness of 10 μm. Before staining, sections need to be put in a fume hood and dry by wind, then washed 3 times in 1× PBS for 5 min each. Subsequently, the sections were blocked with 5% donkey serum in PBST and incubated with appropriately diluted primary antibodies at 4 °C overnight. Afterward, the sections were washed 3 times in 1× PBS for 5 min each and then incubated with suited secondary antibodies or other dyes at room temperature for 40 min in dark. After 3 times 1× PBS washing, the fixed and stained sections were mounted with 50% glycerol and nail polishing oil on four edges of the glass coverslip for further analysis. Alexa fluorescence secondary antibodies were used to develop signals. To amplify weak signals, horseradish peroxidase-conjugated antibodies were used. Immunostaining images were acquired by Zeiss confocal microscopy system (LSM710) and Olympus Laser scanning confocal microscope (Fluoview 1200). The ImageJ (NIH) and Photoline (21.00) software were used to analyze images. The primary antibodies used for IF staining were as follows: F4/80 (Abcam, ab6640; 1:500), GATA6 (Cell Signaling Technology, D61E4; 1:500), CD11b (ThermoFisher Scientific, 14-0112-82; 1:400), tdTomato (Rockland, 600-401-379; 1:1000), CD45 (eBioscience, 17-0451-82; 1:400), CLEC4F (R&D, AF2784; 1:500) and CCR2 (R&D, FAB5538A-100; 1:500). The secondary antibodies were used as follows, Alexa donkey anti-rabbit 488 (Invitrogen, A21206; 1:1000), Alexa donkey anti rabbit 555 (Invitrogen, A31572; 1:1000), Alexa donkey anti-rabbit 647 (Invitrogen, A31573; 1:1000), Alexa donkey anti-rat 488 (Invitrogen, A21208; 1:1000), Alexa donkey anti-rat 647 (Abcam, ab150155; 1:1000), Alexa donkey anti-goat 488 (Invitrogen, A11055; 1:1000), Alexa donkey anti-goat 647 (Invitrogen, A21447; 1:1000), and Impress goat-anti rat (Vector lab, MP-7444; 1:3).

### Cell isolation and flow cytometry

For pleural and peritoneal cavity cells: Mice were anesthetized by hypodermic injection with 1% pentobarbital sodium. Peritoneal cells were isolated by flushing the peritoneal cavity with a single injection of 8 ml sterile cold PBS^58^. For the pleural cavity, 4 ml sterile cold PBS was fine^16,17^. The retracted cell fluid was centrifuged at 500*g* for 5 min at 4 °C and washed with PBS before staining.

For blood cells: Mouse blood was collected into heparin-containing PBS solution. After red blood cell lysing, the cells were washed twice with PBS before staining.

For liver: After killing of the mice, liver biopsies of the injured area were harvested into cold HBSS (Invitrogen, 14026126) with 0.05% collagenase type IV (Worthington, LS004188) after being perfused in vivo via the portal vein with 30 ml HBSS. Then the liver was minced into small pieces and digested by 10 ml HBSS containing 0.05% collagenase Type IV and DNase I (60 U/ml, Worthington, LS002139) at 37 °C, shaking for 30 min. The liver specimen was filtered through a 70 μm cell strainer. Next, the cells were centrifuged at 50*g* for 1 min at 4 °C to collect non-parenchymal cell-enriched supernatant. Then the non-parenchymal cells were purified using centrifugation 33% Percoll (Sigma, P1644) solution containing 10 U/ml heparin (Sigma, H3149). After spinning at 500*g* for 15 min at 4 °C, 1 ml Red Blood Cell lysis buffer (eBioscience, 00-4333-57) was added for 5 min at room temperature. To stop the reaction, 9 ml cold PBS was added and centrifuged at the speed of 500*g* for 5 min at 4 °C to discard the supernatant. After red blood cell lysing, the cells were washed twice with PBS before staining.

For lung: After killing, the mice were perfused with 10 ml cold PBS through the right ventricle to flush out blood cells in the lung. Then the mice were inflated through the trachea with 2 ml digestion solution (Collagenase IV 2 mg/ml, FBS 5% and DNase I 60 U/ml in RPMI-1640 Media. FBS, Gibco, 10099141; RPMI-1640, Invitrogen, 22400089). The lungs were removed and minced into small pieces in 10 ml digestion solution for 30 min at 37 °C with shaking and frequent agitation. After digestion, the cells were filtered through a 70 μm strainer, centrifuged at 500*g* for 15 min at 4 °C to discard the supernatant. Next, cells were incubated in 1 ml Red Blood Cell lysis buffer (eBioscience, 00-4333-57) at room temperature for 5 min. Totally, 9 ml PBS was added and centrifuged at the speed of 500*g* for 5 min at 4 °C to discard the supernatant. After red blood cell lysing, the cells were washed twice with PBS before staining.

For cavity cells, blood cells, liver, and lung single-cell suspension, the cells were stained with primary antibodies containing CD45 FITC (eBioscience, 11-0451, 1:200), F4/80 PE-Cy7 (Biolegend, 123114, 1:200), and CD11b APC (eBioscience, 17-0112-81, 1:200) at 4 °C for 30 min. Next, the cells were washed and re-suspended by PBS, and then stained with DAPI (Vector Laboratories) at 4 °C for 5 min before FACS. The cells were analyzed using Attune NxT Flow Cytometer (Thermo Fisher Scientific). Data were generated using FlowJo (Tree Star). The gating strategies for flow cytometry data analysis are illustrated in Supplementary Fig. 10.

### Cell culture and IF staining and imaging

Before cell culture, the sterile coverslips were coated with 1% Gelatin (Solarbio, G8061) in the 24-well plate in the cell culture hood for at least 1 h. Then discard the excess liquid and dry the coverslips completely in the cell culture hood. The cavity and blood cells were maintained in Dulbecco’s Modified Eagle’s Medium (DMEM, Invitrogen, 11965092), supplemented with 10% fetal bovine serum (Gibco, 10099141) at 37 °C in the presence of 5% CO_2_. After incubation at 37 °C for 4–6 h, removed the cell culture medium and rinsed the cells 3 times with PBS. The following procedures were carried out using standard procedures.

### Sirius red staining

Sirius red staining was used to determine collagen deposition and indicate fibrotic areas in injury models. This was performed using a standard protocol as described previously^59^. In detail, the tissue cryosections were washed in 1× PBS for 15 min and fixed in 4% paraformaldehyde for 10 min at room temperature. Slides were then washed 3 times in 1× PBS for 5 min each. Next, slides were incubated in Bouins’ solution (5% acetic acid, 9% formaldehyde, and 0.9% picric acid) at room temperature overnight. On the next day, after washing by 1× PBS, slides were incubated in 0.1% Fast Green (Fisher, F-99) for 5 min, then in 0.1% Sirius Red (Direct red 80, Sigma, 0-03035) for 3 min. After 3 times washing in 1× PBS, slides were dehydrated with ethanol and xylene. Before microscopy, slides were mounted with Neutral Balsam Mounting Medium (Sangon Biotech, E675007).

### Injury models

For liver CCl_4_ injury: CCl_4_ (SINOPHARM, 10006418) was dissolved at 1:1 in corn oil and administered by gavage at a single dose of 3.5 ml/kg body weight^16^. For the CCl_4_-induced chronic injury model, CCl_4_ was dissolved at 1:3 in corn oil and administrated intragastrically at a dose of 4 µl/g body weight every 3 days, repeated 10 times. For the control groups, mice received the equivalent amount of corn oil.

For liver heat injury (HI): Sterile inflammation induced by thermal injury in the liver was performed as described previously^60^. In detail, mice were anesthetized with 2% isoflurane gas in a sealed chamber. The abdominal fur was removed carefully and the skin was disinfected with iodine. A midline abdominal incision was made just below the level of the diaphragm to expose the liver. A single focal injury was generated on the surface of the liver with High-Temperature Cautery (WORLD PRECISION INSTRUMENTS, 500392). After that, the skin incisions were closed with suture. For sham groups, mice underwent the same surgical procedure without thermal injury.

For liver cryoinjury (CI): The general surgical process was similar with the liver HI model. After mice were anesthetized with isoflurane, the liver was exposed and damaged by touching with a copper probe precooled in liquid nitrogen for about 20 s. The control mice were treated with the same procedures as in the CI model but without CI.

For liver acetaminophen (APAP) injury: Food was withdrawn 12–15 h prior to treatment with APAP (MCE, HY-66005/CS-2819). The drug was administered by gavage with 300 mg/kg and mice were CO_2_ euthanized 24 h later.

For lung bleomycin injury: Alveolar injury was achieved by intratracheal instillation of bleomycin, as described previously^61^. In detail, bleomycin (Sigma B8416) was dissolved at a concentration of 10 U/ml in sterile PBS (Invitrogen, 10010049) and stored as small aliquots at −80 °C. Before use, the bleomycin was diluted to a working concentration 1 U/ml with PBS. After mice were anesthetized with 1% pentobarbital sodium, 2 U/kg bleomycin was pipetted into the cannula. As the mice breathed bleomycin was inhaled into the lung. The control mice were treated with PBS.

For lung CI: We performed the mice lung CI model according to our lab’s protocol as described previously^54^. In detail, mice were first anesthetized with isoflurane in an airtight environment. Then mice were ventilated and anesthetized via tracheotomy, with the cannula connected to a respiratory machine (Harvard Apparatus) and an anesthetic gas machine (Harvard Apparatus). The left lung was exposed by thoracotomy and damaged by touching with a copper probe precooled in liquid nitrogen for about 10 s. After surgery, mice were kept on the warm bed until they awoke. Sham-operated lungs were only exposed without probe application.

For lung LPS injury: 800 µg LPS (*Escherichia coli 055:B5*; Sigma) was dissolved in 50 µl of sterile PBS (Invitrogen, 10010049). After mice were anesthetized with 1% pentobarbital sodium, LPS was pipetted into the cannula by intratracheal instillation. The control mice were treated with PBS^62^.

### Peritoneal macrophages transfer

For peritoneal macrophages transfer, 3 × 10^6^ whole peritoneal cells from *G6Mø-CreER;R26-tdTomato* mice were transferred intraperitoneally one day prior to HI and CCl_4_ treatment as described previously^16,17^. In detail, the peritoneal cells from *G6Mø-CreER;R26-tdTomato* mice were washed three times with cold 1× PBS. Then the cells were resuspended with 100 µl PBS and transferred to recipients.

### Macrophage depletion

Clodronate liposome (YEASEN, Shanghai, China, from Vrije University, Amsterdam) was injected intraperitoneally 7 days prior to the experiment with 200 µl/mice.

### Detection of serum transaminases ALT and AST

The ALT and AST levels in serum were measured by a kit according to the manufacturer’s protocol (Shensuoyoufu Medical Diagnostic company, Shanghai, China) and analyzed by Infinite M200 Pro (TECAN).

### In vitro analysis of macrophage-mediated phagocytosis

Pleural and peritoneal cells were cultured in 12-well plate at a suitable density for at least 2 h to allow adherence. Latex beads-FITC (Sigma, L1030; cell number: beads = 1:50) was washed 3 times with PBS and finally incubated with FBS at 37 °C for 1 h at a concentration of 1 µl beads:100 µl FBS. At the same time, changed the cell culture medium was changed to FBS-free medium for 1 h at 37 °C. After incubation, the beads were centrifuged at 10,656*g* for 3 min and resuspended with an FBS-free medium. Next, the beads were added to each well equally and analyzed at the indicated time point by IF and FACS.

### In vitro analysis of macrophage polarization

Pleural and peritoneal cells were isolated from mice and cultured in a 12-well plate at 37 °C in the presence of 5% CO_2_ for 3 h. Then, cell polarization into M1 or M2 was performed by stimulation with IFN-γ (50 ng/ml, Peprotech, 315-05) plus LPS (10 ng/ml, Solarbio, L8880) or IL4 (20 ng/ml, Solarbio, P00021), respectively. After 24 h culture, cells were prepared for RNA extraction and qRT-PCR was done to measure the related gene expression level.

### RNA extraction

Cells were harvested for total RNA isolation using Trizol reagent (Invitrogen, 15596018). 1 ml Trizol reagent was used to dissolve each sample and was incubated for 5 mins at room temperature (RT). The samples were centrifuged at 10,656*g* for 5 min at 4 °C, and the supernatant was transferred to a new 1.5 ml Eppendorf tube followed by adding 200 μl chloroform. Then the samples were intensely vortexed for 15–20 s and left for 15 min at RT. They were then centrifuged at 4 °C for 10,656*g* for 15 min and the supernatant transferred to a new 1.5 ml Eppendorf tube. Next, 500 μl of isopropyl alcohol was added and the samples were mixed well and then left to stand at RT for 10 min. Next, the samples were centrifuged at 10,656*g* for 15 min at 4 °C and washed RNA with 75% EtOH twice, then air-dried. Appropriate RNase/DNase-free water was used to solve the RNA.

### Quantitative RT-PCR assay

1 μg total RNA was reverse transcribed to cDNA by TaKaRa PrimeScript™ RT reagent Kit with gDNA Eraser (Perfect Real Time, Takara, RP074A). Two separate steps followed: first, 42 °C 2 min for Genomic DNA elimination reaction; Second, 37 °C for 15 min, 85 °C for 5 s and hold at 4 °C in PCR machine for reverse-transcription reaction. The synthesized cDNA was diluted fivefold with MilliQ water and then stored at −20 °C for further experiments. Quantitative real-time PCR (qRT-PCR) was carried out in ABI Step-one plus instrument (Applied Biosystems) using Invitrogen SYBR Green qRT-PCR reagents (Invitrogen, 4367659). Totally, 10 μl reaction system was set up including 5 μl 2× SYBR Green reagent, 1 μl forward and reverse mixed primers, 1 μl diluted cDNA template, and 3 μl ddH_2_O. The PCR program was set as 95 °C 10 min, 40 repetitions of 95 °C 5 s and 60 °C 30 s. The results were normalized to GAPDH and control was set as 1. All primers are listed in Supplementary Table 1.

### Quantification and statistical analysis

All results were presented as the mean ± SD. Statistical analyses were performed using Student’s *t* test with GraphPad Prism 7 software. Totally, 4–8 mice were included in each group of mice in every experiment. Multiple groups were tested via one-way ANOVA, and comparisons between two groups were performed using Student’s *t* test. *P value of <0.05, **P value of <0.01, ***P value of <0.005, and ****P value of <0.0001 were considered significant.

### Reporting summary

Further information on research design is available in the Nature Research Reporting Summary linked to this article.

## Supplementary information

Supplementary Information

Reporting Summary

## Data Availability

All data that support the findings of this study are provided within the paper and its supplementary information. All additional information is available from the corresponding author upon reasonable request. Source data are provided with this paper.

## Source data

Source Data
