# Supplementary material for: Genetic fate-mapping reveals surface accumulation but not deep organ invasion of pleural and peritoneal cavity macrophages following injury
Source: Nat Commun. 2021 May 17;12:2863. doi: 10.1038/s41467-021-23197-7 (PMC8129080; doi:10.1038/s41467-021-23197-7)
Supplement: Supplementary file 1 — Supplementary Information [file 41467_2021_23197_MOESM1_ESM.pdf]

Supplementary Information for

Genetic fate-mapping reveals surface accumulation but not deep organ invasion of pleural and peritoneal cavity macrophages following injury

Hengwei Jin, Kuo Liu, Juan Tang, Xiuzheng Huang, Haixiao Wang, Qianyu Zhang, Huan Zhu, Yan Li, Wenjuan Pu, Huan Zhao, Lingjuan He, Yi Li, Shaohua Zhang, Zhenqian Zhang, Yufei Zhao, Yanqing Qin, Stefan Pflanz, Karim EI Kasmi, Weiyi Zhang, Zhaoyuan Liu, Florent Ginhoux, Yong Ji, Ben He, Lixin Wang, Bin Zhou*

*Correspondence to: zhoubin@sibs.ac.cn

The following sections include:

Supplementary Figures 1 to 10

Supplementary Table 1

Supplementary Figure 1

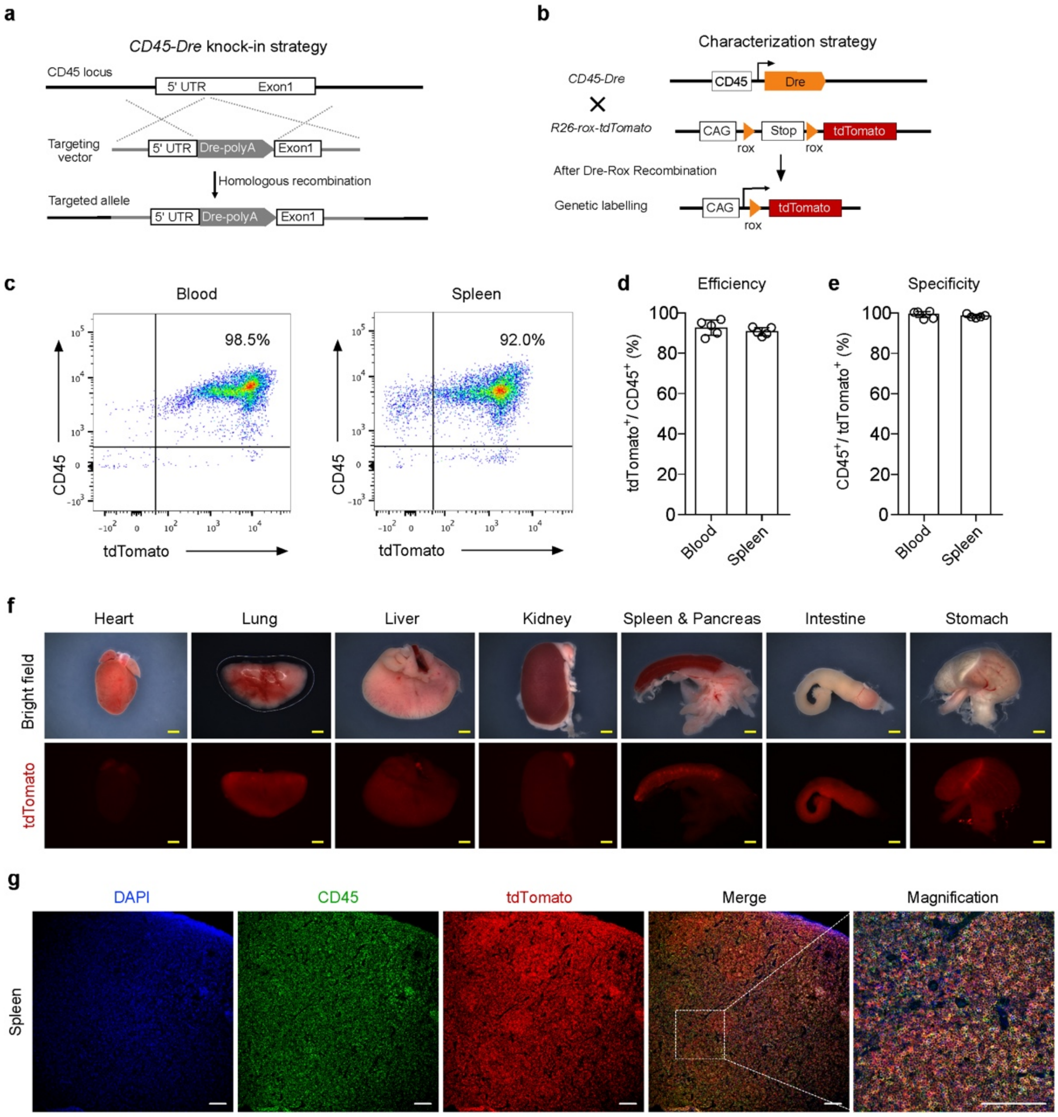

Supplementary Figure 1. Generation and characterization of *CD45-Dre* knock-in allele. Related to Fig.1. **a**, Schematic figure showing knock-in strategy for generation of *CD45-Dre* allele by homologous recombination using CRISPR/Cas9. **b**, *CD45-Dre* mouse was crossed with rox reporter line *R26-rox-tdTomato*. After Dre-rox recombination, tdTomato is expressed in CD45$^+$ cells and their descendants. **c**, Flow cytometric analysis of cells from blood and spleen of *CD45-Dre;R26-rox-tdTomato* mice at 8 weeks. **d**, Quantification of the labeling efficiency by percentage of tdTomato$^+$ cells in CD45$^+$ cells. Data are the mean ± SD; n = 5 mice per group. **e**, Quantification of the labeling specificity by percentage of CD45$^+$ cells in tdTomato$^+$ cells. Data are the mean ± SD; n = 5 mice per group. **f**, Whole-mount bright field and

epifluorescence images of visceral organs. **g**, Immunostaining for tdTomato and CD45 on spleen sections showed that the vast majority of tdTomato$^+$ cells in the spleen were CD45$^+$ cells. Boxed region is magnified. Each image is representative of 5 individual samples. Scale bars, 1 mm in whole-mount pictures; 100 μm in sections. Source data are provided as a Source Data file.

Supplementary Figure 2

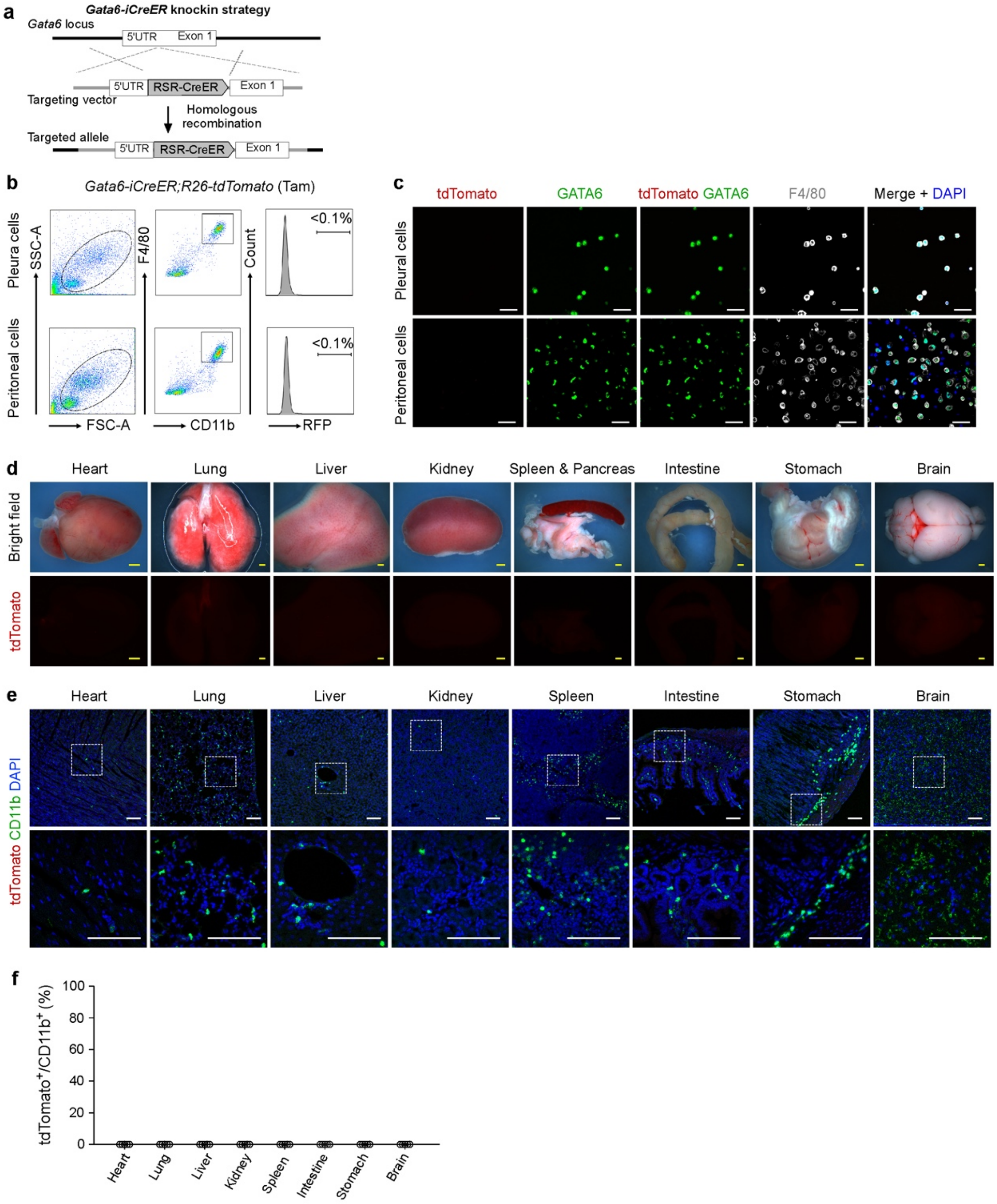

Supplementary Figure 2. *Gata6-iCreER* does not target macrophages or other visceral organs after tamoxifen induction. Related to Fig.1. **a**, Schematic figure showing knockin strategy using CRISPR/Cas9. **b**, Flow cytometric analysis of percentage of RFP^+ cells in pleural and peritoneal macrophages. Tamoxifen (Tam) was treated at week 8 and tissues were collected one week later. **c**, Immuostaining for tdTomato, GATA6, and F4/80 on dissociated pleural or peritoneal cells. **d**, Whole-mount bright field or epifluorescence images of visceral organs. **e**, Immunostaining for

tdTomato and CD11b on tissue sections. Boxed regions are magnified. **f**, Quantification of the percentage of $CD11b^+$ cells expressing tdTomato. Data are the mean ± SD; n = 5 mice per group. Scale bars: yellow, 1 mm; white, 100 μm. Each image is representative of 5 individual biological samples. Source data are provided as a Source Data file.

Supplementary Figure 3

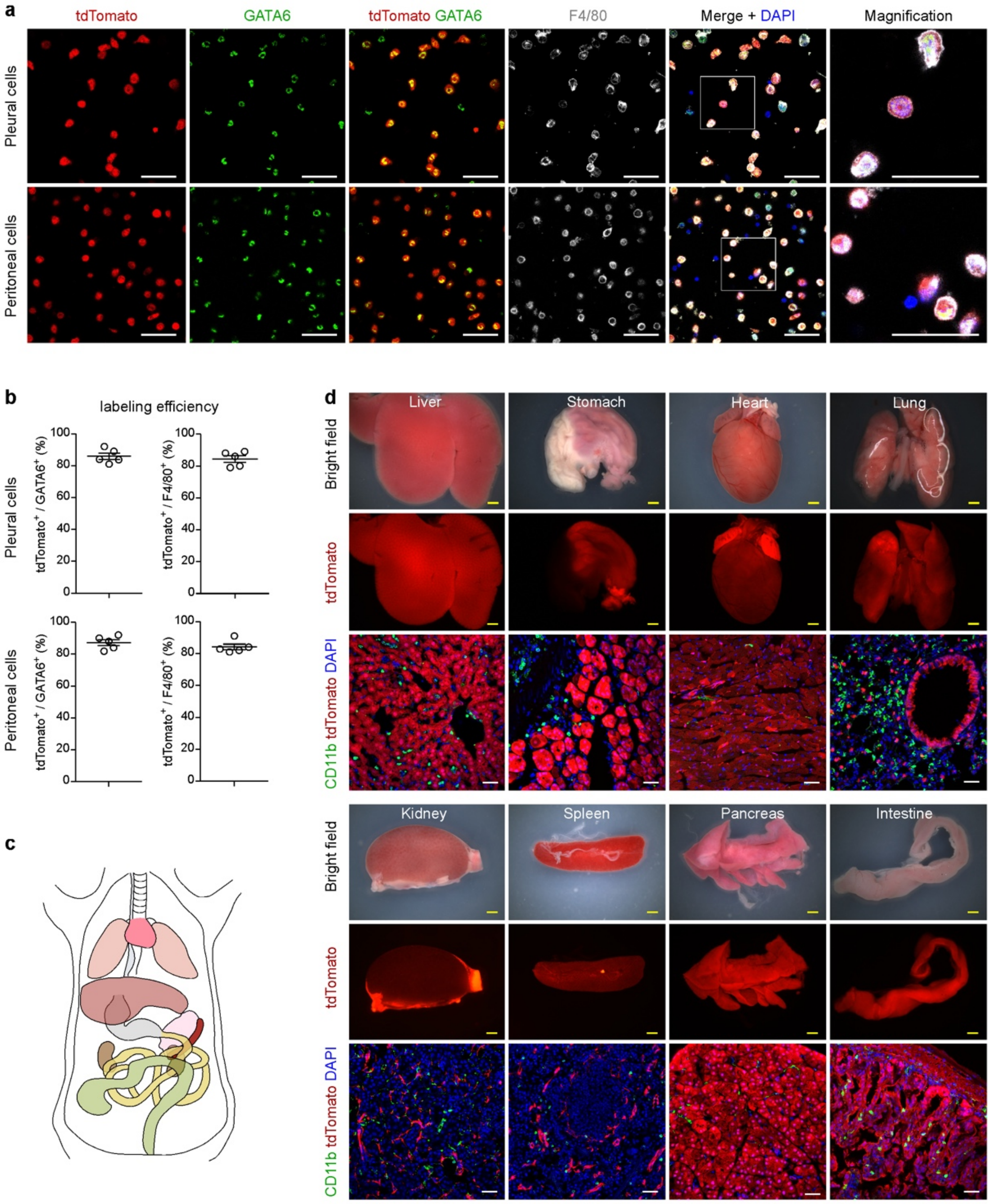

Supplementary Figure 3. ***Gata6-CreER*** **targets visceral organs broadly in addition to peritoneal and pleural macrophages.** Related to Fig.1. **a**, Immunostaining for tdTomato, GATA6 and F4/80 on isolated pleural cells or peritoneal cells. Mice were treated with tamoxifen at 8 weeks and tissues were collected one week later. Boxed regions are magnified. **b**, Quantification of the labeling efficiency of GATA6^{+} or F4/80^{+} macrophages from pleural and peritoneal cells. Data are the mean ± SD; n = 5 mice per group. **c**, Cartoon figure showing visceral organs collected for examination. **d**, Whole-mount bright field and epifluorescence images of visceral organs, and

immunostaining for tdTomato and CD11b on tissue sections showed that the vast majority of tdTomato^{+} cells in visceral organs are not macrophages. Scale bars: yellow, 1 mm; white, 100 μm. Each image is representative of 5 individual samples. Source data are provided as a Source Data file.

Supplementary Figure 4

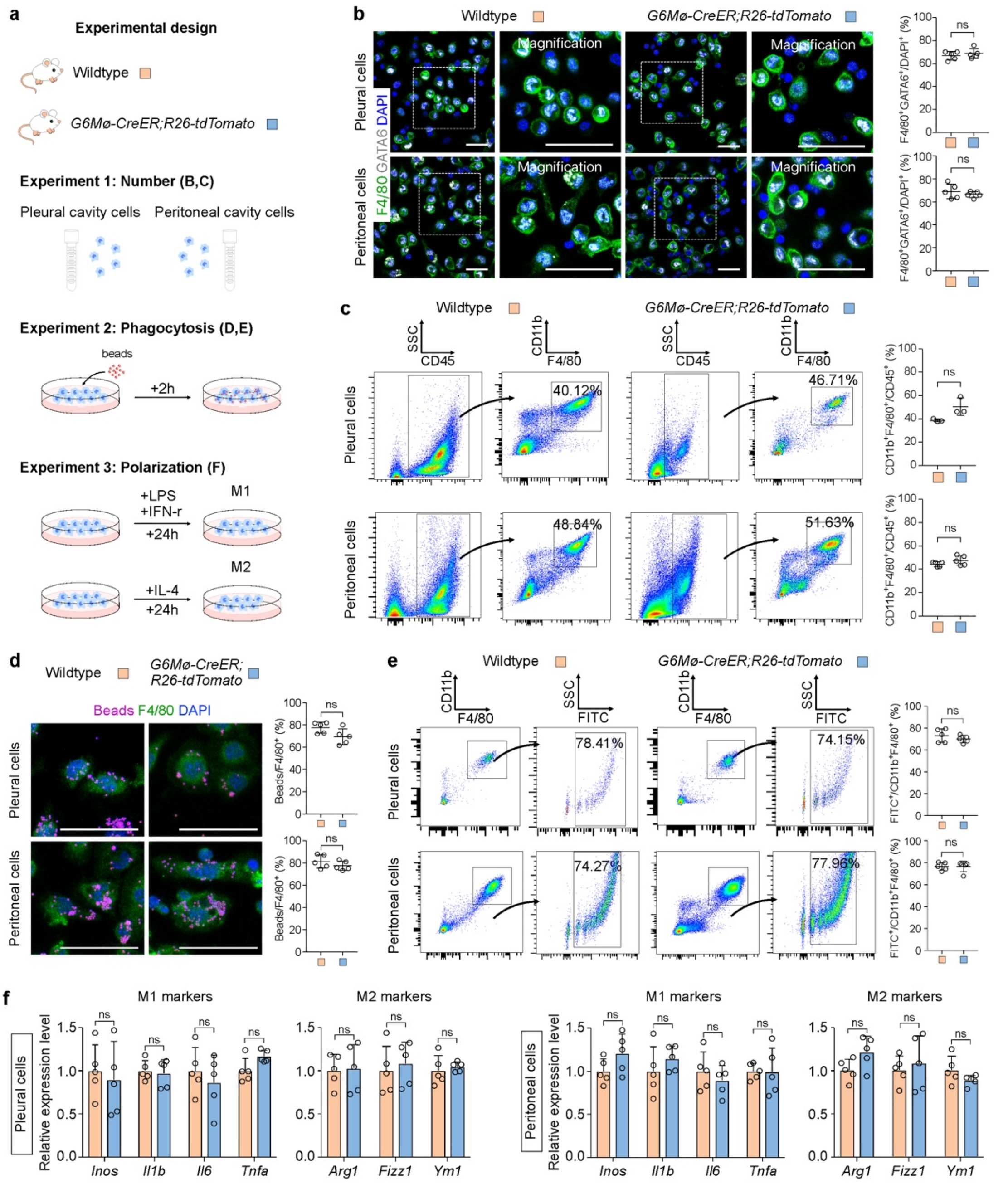

Supplementary Figure 4. *Gata6* heterozygosity does not affect number and function cavity macrophage. Related to Fig.6. **a**, Schematic figure showing experimental strategy. **b**, Immunostaining for F4/80 and GATA6 on dissociated cells from pleural or peritoneal cavity. Boxed regions are magnified. Scale bars, 100 μm. Right panel show quantification of the percentage of GATA6$^+$F4/80$^+$ macrophages in all cavity cells. Data are the mean ± SD; n = 5 mice per group; ns, non-significant. **c**, FACS analysis and quantification of the percentage of CD11b$^+$F4/80$^+$ cells in CD45$^+$ cells from pleural and peritoneal cavity. n = 5 mice per group. ns, non-significant. **d**,

Immunostaining for F4/80 on pleural and peritoneal cells treated with FBS-coated beads. Scale bars, 100 μm. Right panels show quantification of the percentage of phagocytic F4/80$^+$ cells in all F4/80$^+$ macrophages. Data are the mean ± SD; n = 5 mice per group. ns, non-significant. **e**, FACS analysis and quantification of pleural and peritoneal macrophages express FITC. Data are the mean ± SD; n = 5 mice per group. ns, non-significant. **f**, Measurement of mRNA expressions of M1/M2 polarization genes in pleural and peritoneal macrophages in WT and *Gata6* heterozygous group. Data are the mean ± SD; n = 5 mice per group. ns, non-significant. *P* value was calculated by unpaired two-sided Student's *t*-test (b-f). Each image is representative of 5 individual samples. Source data are provided as a Source Data file.

Supplementary Figure 5

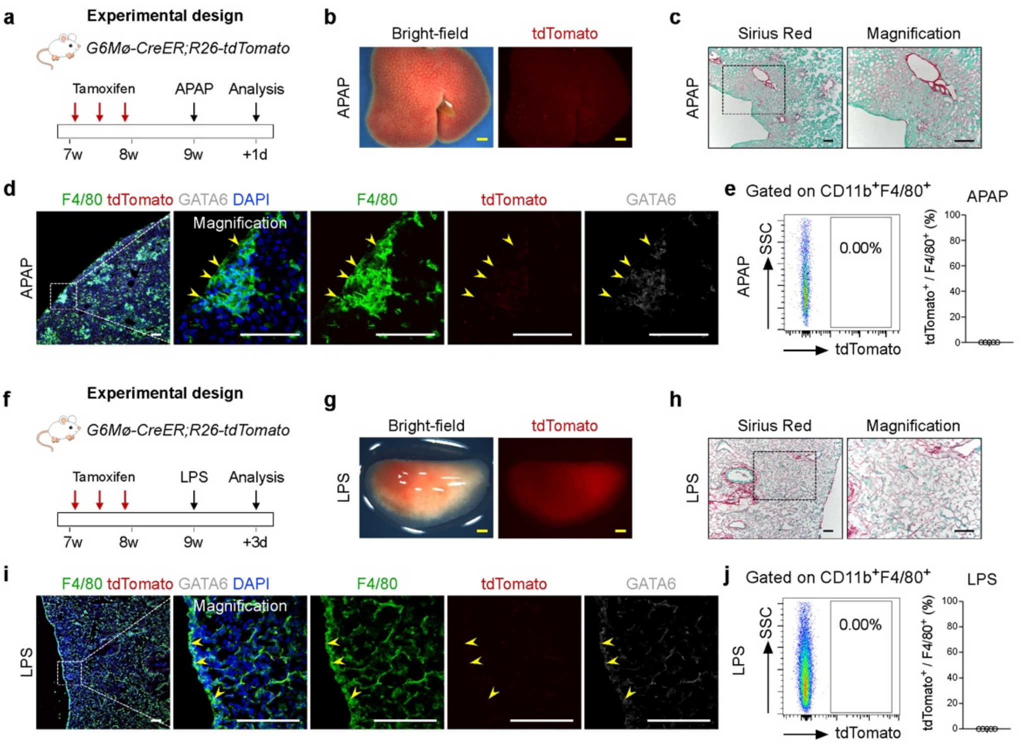

Supplementary Figure 5. No accumulation of cavity macrophages on APAP-induced liver injury or LPS-induced lung injury. Related to Fig.2 and Fig.3. **a**, Schematic figure showing experimental strategies. APAP, acetaminophen. **b**, Whole-mount bright-field and fluorescent images of liver after APAP treatment. Scale bars, 1 mm. **c**, Sirius red staining of liver tissue sections after APAP treatment. Boxed region is magnified. Scale bars, 100 μm. **d**, Immunostaining for tdTomato, GATA6 and F4/80 on injured regions of liver. Arrowheads, injury site. Scale bars, 100 μm. **e**, FACS and quantification analysis of the percentage of macrophages expressing tdTomato from injury regions of liver. Data are the mean ± SD; n = 5 mice per group. Each figure is representative of 5 individual biological samples. **f**, Schematic figure showing experimental strategies. LPS, lipopolysaccharide. **g**, Whole-mount bright-field and fluorescent images of lung after LPS treatment. Scale bars, 1 mm. **h**, Sirius red staining of lung tissue sections after LPS treatment. Boxed region is magnified. Scale bars, 100 μm. **i**, Immunostaining for tdTomato, GATA6 and F4/80 on injured regions of lung. Arrowheads, injury site. Scale bars, 100 μm. **j**, FACS and quantification analysis of the percentage of macrophages expressing tdTomato from injury regions of lung. Data are

the mean ± SD; n = 5 mice per group. Each image is representative of 5 individual biological samples. Source data are provided as a Source Data file.

Supplementary Figure 6

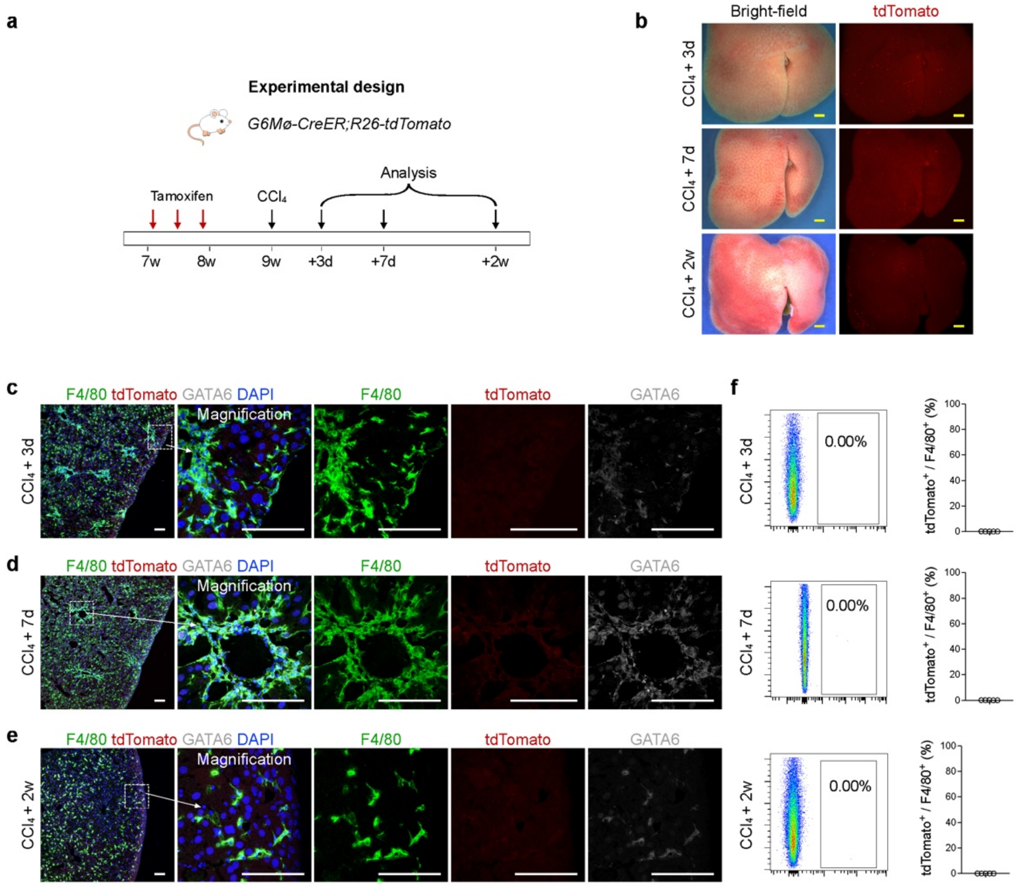

Supplementary Figure 6. Peritoneal cavity macrophages do not invade deep into the tissue during later injury phases. Related to Fig.2. **a**, Schematic figure showing experimental strategies. **b**, Whole-mount bright-field and fluorescent images of livers at 3 days, 7 days, and 2 weeks after CCl_4-induced liver injury. Scale bars, 1 mm. **c-e**, Immunostaining for tdTomato, GATA6 and F4/80 on injured regions of livers at different time point. Boxed regions are magnified. Scale bars, 100 μm. **f**, FACS and quantification analysis of the percentage of macrophages expressing tdTomato from injury regions of livers at different time point. Data are the mean ± SD; n = 5 mice per group. Each image is representative of 5 individual biological samples. Source data are provided as a Source Data file.

Supplementary Figure 7

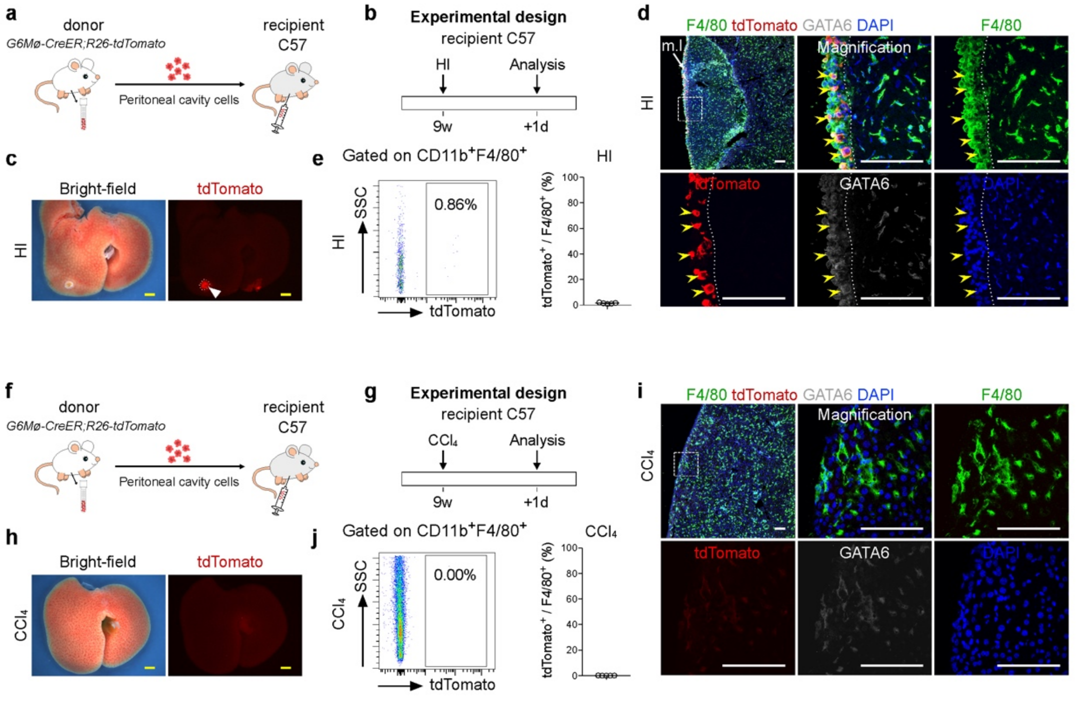

Supplementary Figure 7. Peritoneal macrophages do not invade deep into the liver by adoptive transfer. Related to Fig.2. **a**, Peritoneal cavity cells were harvested from *G6Mø-CreER;R26-tdTomato* mice and transplanted to C57 wild-type mice. **b**, Schematic figure showing experimental strategy. HI, heat injury. **c**. Whole-mount bright-field and fluorescent images of livers with HI injury. Arrowhead, injury site. Scale bars, 1 mm. **d**, Immunostaining for tdTomato, GATA6, and F4/80 on injured region of liver section. m.l., mesothelial layer. Arrowheads, injury site. Scale bars, 100 µm. **e**, FACS analysis and quantification of the percentage of $F4/80^+$ macrophages expression tdTomato from injury regions of livers. Data are the mean ± SD; n = 5 mice per group. **f**, Peritoneal cavity cells were harvested from *G6Mø-CreER;R26-tdTomato* mice and transplanted to C57 wild-type mice. **g**, Schematic figure showing experimental strategy. **h**. Whole-mount bright-field and fluorescent images of livers with CCl_4 injury. Scale bars, 1 mm. **i**, Immunostaining for tdTomato, GATA6, and F4/80 on injured region of liver section. Boxed region is magnified. Scale bars, 100 µm. **j**, FACS and quantification analysis of the percentage of macrophages expression tdTomato. Data are the mean ± SD; n = 5 mice per group. Each image is representative of 5 individual samples. Source data are provided as a Source Data file.

Supplementary Figure 8

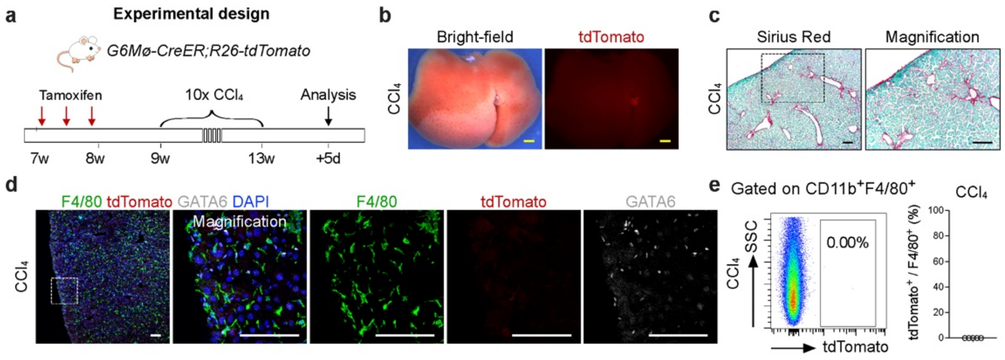

Supplementary Figure 8. Peritoneal cavity macrophages do not invade deep into the tissue in CCl_4-induced chronic liver injury. Related to Fig.2. **a**, Schematic figure showing experimental strategies. **b**, Whole-mount bright-field and fluorescent images of liver after CCl_4 treatment. Scale bars, 1 mm. **c**, Sirius red staining of liver tissue sections after CCl_4 in liver. Boxed region is magnified. Scale bars, 100 µm. **d**, Immunostaining for tdTomato, GATA6 and F4/80 on injured regions of liver. Boxed region is magnified. Scale bars, 100 µm. **e**, FACS and quantification analysis of the percentage of macrophages expressing tdTomato from injury regions of liver. Data are the mean ± SD; n = 5 mice per group. Each image is representative of 5 individual biological samples. Source data are provided as a Source Data file.

Supplementary Figure 9

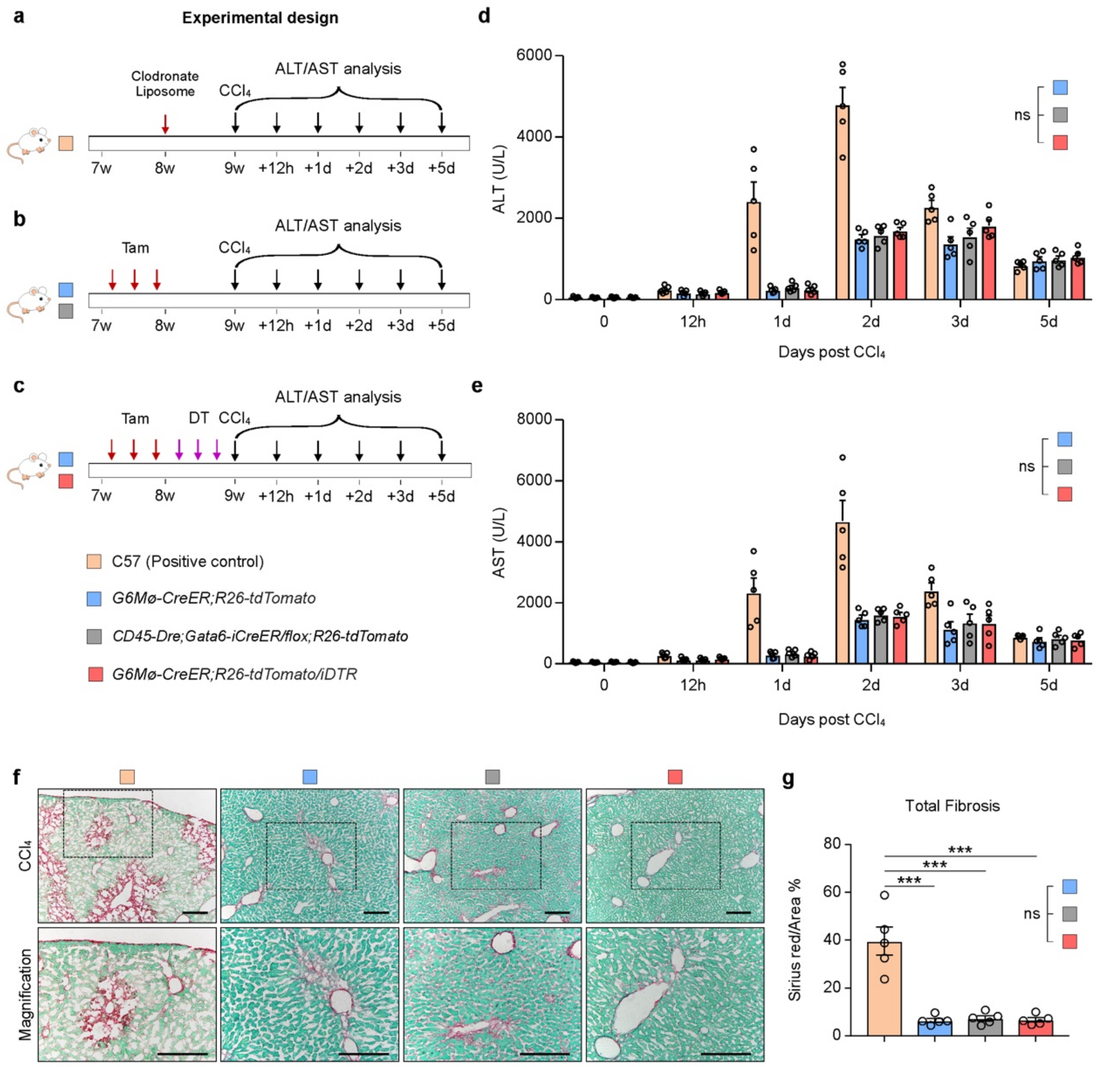

Supplementary Figure 9. Functional analysis of livers reveals no significant difference after knockout of *Gata6* or ablation of cavity macrophages. Related to Fig.4 and Fig.5. **a-c**, Schematic figure showing experimental strategies. DT, diphtheria toxin. ALT, Alanine aminotransferase. AST, Aspartate aminotransferase. **d, e,** Time course of ALT and AST activities in serum from different mice groups after CCl_4 treatment. ns, non-significant. Data are the mean ± SD; n = 5 mice per group. **f**, Representative sirius red stained liver sections from different mice groups of 5 days after CCl_4 treatment. Boxed regions are magnified. Scale bars, 100 μm. **g**, Quantification analysis of the fibrotic areas of livers after injury. ns, non-significant. ns, non-significant. Data are the mean ± SD; n = 5 mice per group. ****P* = 0.0005 in C57 vs. *G6Mø-CreER;R26-tdTomato*. ****P* = 0.0007 in C57 vs. *CD45-Dre;Gata6-*

iCreER/flox;R26-tdTomato. ****P* = 0.0006 in C57 vs. *G6Mø-CreER;R26-tdTomato/iDTR*. *P* value was calculated by unpaired two-sided Student's *t*-test (g) or two-way ANOVA coupled with multiple comparisons (d, e). Each figure is representative of 5 individual biological samples. Source data are provided as a Source Data file.

Supplementary Figure 10

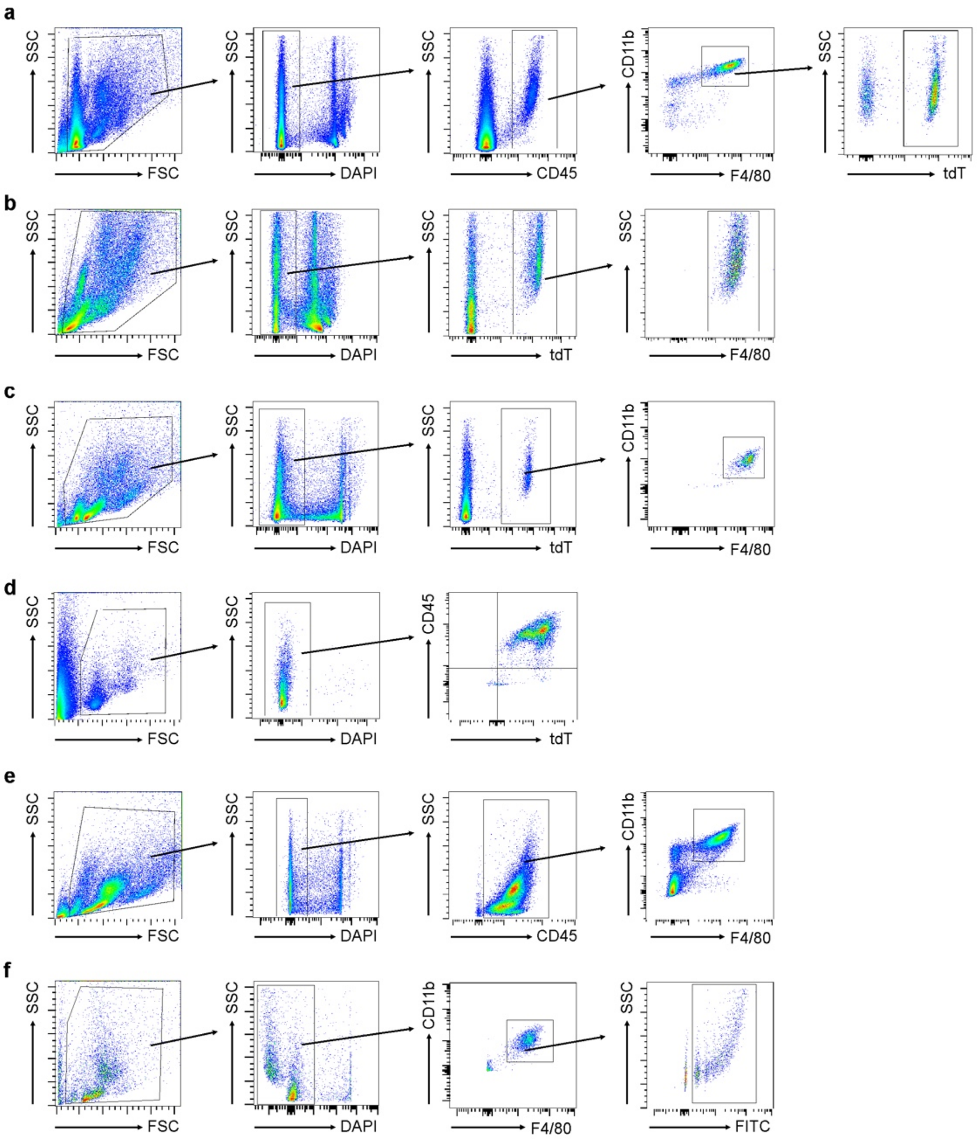

Supplementary Figure 10. Gating strategy for FACS. a, Gating strategy to analyze the percentage of tdTomato$^+$ cells in macrophages (CD45$^+$CD11b$^+$F4/80$^+$). For Fig. 1d, Fig. 2e, Fig. 3e, Fig. 4b, d, Fig. 5b, e, Fig. 6d, Supplementary Fig. 2b, Supplementary Fig. 5e, j, Supplementary Fig. 6f, Supplementary Fig. 7e, j and Supplementary Fig. 8e. **b**, Gating strategy to analyze the percentage of macrophages (F4/80$^+$) in tdTomato$^+$ cells. For Fig. 1h. **c**, Gating strategy to analyze the percentage of macrophages (CD11b$^+$F4/80$^+$) in tdTomato$^+$ cells. For Fig. 6e. **d**, Gating strategy to analyze the percentage of tdTomato$^+$ cells in CD45$^+$ cells. For Supplementary Fig. 1c**. e**, Gating strategy to analyze the percentage of macrophages (CD11b$^+$F4/80$^+$) in CD45$^+$ cells. For Supplementary Fig. 4c. **f**, Gating strategy to analyze the percentage of beads (FITC$^+$)

in macrophages ($CD11b^+F4/80^+$). For Supplementary Fig. 4e.

Supplementary Table 1

Primers used for quantitative PCR.

Gene	Forward	Reverse
Gapdh	CCTTCCGTGTTCCTACCCC	GCCCAAGATGCCCTTCAGT
Tnfa	CTGAACTTCGGGGTGATCGG	GGCTTGTCACTCGAATTTTGAGA
Il1b	GAAATGCCACCTTTTGACAGTG	CTGGATGCTCTCATCAGGACA
Il6	CTGCAAGAGACTTCCATCCAG	AGTGGTATAGACAGGTCTGTTGG
Inos	ACATCGACCCGTCCACAGTAT	CAGAGGGGTAGGCTTGTCTC
Arg1	TTGGGTGGATGCTCACACTG	TTGCCCATGCAGATTCCC
Ym1	TTATCCTGAGTGACCCTTCTAAG	TCATTACCCTGATAGGCATAGG
Fizz1	TACTTGCAACTGCCTGTGCTTACT	TATCAAAGCTGGGTTCTCCACCTC
