# Supplementary material for: Genetic fate-mapping reveals surface accumulation but not deep organ invasion of pleural and peritoneal cavity macrophages following injury
Source: Nat Commun. 2021 May 17;12:2863. doi: 10.1038/s41467-021-23197-7 (PMC8129080; doi:10.1038/s41467-021-23197-7)
Supplement: Supplementary file 2 — Reporting Summary [file 41467_2021_23197_MOESM2_ESM.pdf]

nature research

Corresponding author(s): Bin Zhou

Last updated by author(s): Mar 8, 2021

Reporting Summary

Nature Research wishes to improve the reproducibility of the work that we publish. This form provides structure for consistency and transparency in reporting. For further information on Nature Research policies, see our Editorial Policies and the Editorial Policy Checklist.

Statistics

For all statistical analyses, confirm that the following items are present in the figure legend, table legend, main text, or Methods section.

n/a	Confirmed	
☐	☒	The exact sample size (*n*) for each experimental group/condition, given as a discrete number and unit of measurement
☐	☒	A statement on whether measurements were taken from distinct samples or whether the same sample was measured repeatedly
☐	☒	The statistical test(s) used AND whether they are one- or two-sided<br>*Only common tests should be described solely by name; describe more complex techniques in the Methods section.*
☒	☐	A description of all covariates tested
☒	☐	A description of any assumptions or corrections, such as tests of normality and adjustment for multiple comparisons
☐	☒	A full description of the statistical parameters including central tendency (e.g. means) or other basic estimates (e.g. regression coefficient) AND variation (e.g. standard deviation) or associated estimates of uncertainty (e.g. confidence intervals)
☐	☒	For null hypothesis testing, the test statistic (e.g. *F*, *t*, *r*) with confidence intervals, effect sizes, degrees of freedom and *P* value noted<br>*Give P values as exact values whenever suitable.*
☒	☐	For Bayesian analysis, information on the choice of priors and Markov chain Monte Carlo settings
☒	☐	For hierarchical and complex designs, identification of the appropriate level for tests and full reporting of outcomes
☒	☐	Estimates of effect sizes (e.g. Cohen's *d*, Pearson's *r*), indicating how they were calculated

Our web collection on statistics for biologists contains articles on many of the points above.

Software and code

Policy information about availability of computer code

Data collection	Zeiss stereoscope (AxioZoom V16) was used for whole-mount bright-field and fluorescence images; Zeiss confocal microscopy system (LSM710) and Olympus Laser scanning confocal microscope (Fluoview 1200) were used for immunostaining images; Attune NxT Flow Cytometer (Thermo Fisher Scientific) was used for FACS; ABI Step-one plus instrument (Applied Biosystems) was used for quantitative RT-RCP; Infinite M200 Pro (TECAN) was used for measuring the levels of ALS and AST in serum.
Data analysis	Image J (FIJI, 2.0.0-rc-69/1.52p) and Photoline (21.00) were used for immunofluorescent and bright-filed images analysis. Flow Jo 10.4 was used for flow cytometry. GraphPad Prism 7 was used for data analysis.

For manuscripts utilizing custom algorithms or software that are central to the research but not yet described in published literature, software must be made available to editors and reviewers. We strongly encourage code deposition in a community repository (e.g. GitHub). See the Nature Research guidelines for submitting code & software for further information.

Data

Policy information about availability of data

All manuscripts must include a data availability statement. This statement should provide the following information, where applicable:

- Accession codes, unique identifiers, or web links for publicly available datasets
- A list of figures that have associated raw data
- A description of any restrictions on data availability

All data that support the findings of this study are provided within the paper and its supplementary information. All additional information is available from the corresponding author upon reasonable request. Source data are provided with this paper.

Field-specific reporting

Please select the one below that is the best fit for your research. If you are not sure, read the appropriate sections before making your selection.

☒ Life sciences ☐ Behavioural & social sciences ☐ Ecological, evolutionary & environmental sciences

For a reference copy of the document with all sections, see nature.com/documents/nr-reporting-summary-flat.pdf

Life sciences study design

All studies must disclose on these points even when the disclosure is negative.

Sample size	For all experiments, n≥3 was used according to standard scientific conventions and previous published papers of similar experiments (Deniset et al., Immunity, 2019; Tian et al., Nat Commun, 2017; Wang and Kubes, Cell, 2016). For details, each sample size were described in each figure legend.
Data exclusions	No data were excluded from consideration.
Replication	All reported results in this study were repeated at least 5 times to confirm the reproducibility of the findings. All replication attempts were successful.
Randomization	Male and female mice at the defined age were randomly assigned to different experiment groups.
Blinding	For data collection and analysis, the investigatators were blinded to the allocation of different experiments.

Reporting for specific materials, systems and methods

We require information from authors about some types of materials, experimental systems and methods used in many studies. Here, indicate whether each material, system or method listed is relevant to your study. If you are not sure if a list item applies to your research, read the appropriate section before selecting a response.

Materials & experimental systems

n/a	Involved in the study	
☐	☒	Antibodies
☒	☐	Eukaryotic cell lines
☒	☐	Palaeontology and archaeology
☐	☒	Animals and other organisms
☒	☐	Human research participants
☒	☐	Clinical data
☒	☐	Dual use research of concern

Methods

n/a	Involved in the study	
☒	☐	ChIP-seq
☐	☒	Flow cytometry
☒	☐	MRI-based neuroimaging

Antibodies

Antibodies used

For immunostaining, F4/80 (Abcam, ab6640; 1:500), GATA6 (Cell Signaling Technology, D61E4; 1:500), CD11b (ThermoFisher Scientific, 14-0112-82; 1:400), tdTomato (Rockland, 600-401-379; 1:1000), CD45 (eBioscience, 17-0451-82; 1:400), CLEC4F (R&D, AF2784; 1:500) and CCR2 (R&D, FAB5538A-100; 1:500) were used as described. The secondary antibodies were as follows, Alexa donkey anti rabbit 488 (Invitrogen, A21206; 1:1000), Alexa donkey anti rabbit 555 (Invitrogen, A31572; 1:1000), Alexa donkey anti rabbit 647 (Invitrogen, A31573; 1:1000), Alexa donkey anti-rat 488 (Invitrogen, A21208; 1:1000), Alexa donkey anti-rat 647 (Abcam, ab150155; 1:1000), Alexa donkey anti-goat 488 (Invitrogen, A11055; 1:1000), Alexa donkey anti-goat 647 (Invitrogen, A21447; 1:1000) and Immpress goat-anti rat (Vector lab, MP-7444; 1:3). For FACS, CD45 FITC (eBioscience, 11-0451, 1:200), F4/80 PE-Cy7 (Biolegend, 123114, 1:200) and CD11b APC (eBioscience, 17-0112-81, 1:200) were used as described.

Validation

These antibodies were all commercially available. The species and application of all the antibodies have been appropriately validated by manufacturers and provided on the website and datasheets as follows:
F4/80 (Abcam, ab6640), PMID: 31700166, PMID:31719643, PMID:31949138;
GATA6 (Cell Signaling Technology, D61E4), PMID:32246014,PMID: 32127042,PMID: 31626770;
CD11b (ThermoFisher Scientific, 14-0112-82), PMID: 30333307, PMID: 29866139, PMID: 30417077;
tdTomato (Rockland, 600-401-379), PMID: 32332079, PMID: 31901081, PMID: 31993055;
CD45 (eBioscience, 17-0451-82), PMID: 21502490, PMID: 29755322, PMID: 29163469;
CLEC4F (R&D, AF2784), PMID: 31291357, PMID: 29717162;
CCR2 (R&D, FAB5538A-100), PMID: 32739869, PMID: 32310998, PMID: 31042769;
Alexa donkey anti rabbit 488 (Invitrogen, A21206; 1:1000), PMID: 30646897, PMID: 27715385, PMID: 27723457;
Alexa donkey anti rabbit 555 (Invitrogen, A31572; 1:1000), PMID: 31934347, PMID: 31771978, PMID: 27815415;
Alexa donkey anti rabbit 647 (Invitrogen, A31573; 1:1000), PMID: 27941801, PMID: 27882946, PMID: 30531936;
Alexa donkey anti-rat 488 (Invitrogen, A21208; 1:1000), PMID: 31665628, PMID: 28984244, PMID: 28349968;

Alexa donkey anti-rat 647 (Abcam, ab150155; 1:1000), PMID: 32001778, PMID: 31165579, PMID: 30973939;
Alexa donkey anti-goat 488 (Invitrogen, A11055; 1:1000), PMID: 27815415, PMID: 30851734, PMID: 28349968;
Alexa donkey anti-goat 647 (Invitrogen, A21447; 1:1000), PMID: 24481605, PMID: 27513343, PMID: 28125268;
Immpress goat-anti rat (Vector lab, MP-7444; 1:3), PMID: 31771978, PMID: 28608850, PMID: 28167652;
CD45 FITC (eBioscience, 11-0451), PMID: 28405519, PMID: 32226299, PMID: 31787973;
F4/80 PE-Cy7 (Biolegend, 123114), PMID: 18372338, PMID: 19509298, PMID: 23554311;
CD11b APC (eBioscience, 17-0112-81), PMID: 28638744, PMID: 28405524, PMID: 27757304;

Animals and other organisms

Policy information about studies involving animals; ARRIVE guidelines recommended for reporting animal research

Laboratory animals	7- to10-week-old C57BL/6J genetic background mice (both male and female) were used in this study: CD45-Dre, R26-iDTR, CAG-Dre, R26-tdTomato, R26-rox-tdTomato, Gata6-iCreER, Gata6-iCreER2, Ms4a3-CreER and Gata6-flox mice.C57BL/6l mice were also used as recipients. All mice were kept in group housing (2-5 mice per cage) in a specific pathogen-free facility with controlled environmental conditions of temperature (20-25°C), humidity (30-70%) and lighting (a 12-h light/dark cycle) at Center for Excellence in Molecular Cell Science, Chinese Academy of Sciences.
Wild animals	No wild animals were used in this study.
Field-collected samples	No field-collected samples were included in this study.
Ethics oversight	All animal protocols used in this study were approved by the experimental animal facility which has been accredited by the Institutional Animal Care and Use Committee (IACUC) of the State Key Laboratory of Cell Biology, Shanghai Institute of Biochemistry and Cell Biology, Center for Excellence in Molecular Cell Science, University of Chinese Academy of Sciences, Chinese Academy of Sciences.

Note that full information on the approval of the study protocol must also be provided in the manuscript.

Flow Cytometry

Plots

Confirm that:

- [x] The axis labels state the marker and fluorochrome used (e.g. CD4-FITC).
- [x] The axis scales are clearly visible. Include numbers along axes only for bottom left plot of group (a 'group' is an analysis of identical markers).
- [x] All plots are contour plots with outliers or pseudocolor plots.
- [x] A numerical value for number of cells or percentage (with statistics) is provided.

Methodology

Sample preparation	For pleural and peritoneal cavity cells: Mice were anesthetized by hypodermic injection with 1% pentobarbital sodium. Peritoneal cells were isolated by flushing the peritoneal cavity with a single injection of 8ml sterile cold PBS. For pleural cavity, 4 ml sterile cold PBS was fine. The retracted cell fluid was centrifuged at 500 g for 5 min at 4°C and washed with PBS before staining.<br>For blood cells: Mouse blood was collected into heparin-containing PBS solution. After red blood cell lysing, the cells were washed twice with PBS before staining.<br>For Liver: After killing of the mice, liver biopsies of the injured area were harvested into cold HBSS with 0.05% collagenase type IV (Worthington) after being perfused in vivo via the portal vein with 30ml HBSS. Then the liver was minced into small pieces and digested by 10 ml HBSS containing 0.05% collagenase Type IV and DNase I (60 U/ml) at 37°C, shaking for 30 min. The liver specimen was filtered through 70 µm cell strainer. Next, the cells were centrifuged at 50 g for 1 min at 4°C to collect non-parenchymal cell-enriched supernatant. Then the non-parenchymal cells were purified using centrifugation 33% Percoll solution containing 10U/ml heparin. After spinning at 500x g for 15 min at 4°C, 1 ml Red Blood Cell lysis buffer (eBioscience, 00-4333-57) was added for 5 min at room temperature. To stop reaction, 9ml cold PBS was added and centrifuged at the speed of 500x g for 5 min at 4°C to discard supernatant. After red blood cell lysing, the cells were washed twice with PBS before staining.<br>For Lung: After killing, the mice were perfused with 10 ml cold PBS through the right ventricle to flush out blood cells in the lung. Then the mice were inflated through the trachea with 2 ml digestion solution (Collagenase IV 2 mg/ml, FBS 5% and DNase I 60 U/ml in RPMI-1640 Media). The lungs were removed and minced into small pieces in 10 ml digestion solution for 30 min at 37°C with shaking and frequent agitation. After digestion, the cells were filtered through a 70 µm strainer, centrifuged at 500x g for 15 min at 4°C to discard supernatant. Next, cells were incubated in 1 ml Red Blood Cell lysis buffer (eBioscience, 00-4333-57) at room temperature for 5 min. 9 ml PBS was added and centrifuged at the speed of 500x g for 5 min at 4°C to discard supernatant. After red blood cell lysing, the cells were washed twice with PBS before staining.
Instrument	The cells were analyzed using Attune NxT Flow Cytometer (Thermo Fisher Scientific).
Software	Data were generated using FlowJo 10.4 (Tree Star).
Cell population abundance	Cells for FACS were over 100,000.

Gating strategy

FSC and SSC were used to gate the aimed cell population, and then live cells were gated by Dapi staining. In living cells, Cd11b and F4/80 double positive cells were gated from CD45 positive cells. tdTomato positive cells were gated from Cd11b and F4/80 double positive cells for analysis.

☒ Tick this box to confirm that a figure exemplifying the gating strategy is provided in the Supplementary Information.
